# Engineered Phage-Based Cancer Vaccines: Current Advances and Future Directions

**DOI:** 10.3390/vaccines11050919

**Published:** 2023-04-29

**Authors:** Murali Ragothaman, So Young Yoo

**Affiliations:** BIO-IT Foundry Technology Institute, Pusan National University, Busan 46241, Republic of Korea

**Keywords:** bacteriophages, cancer immunotherapy, phage display, nanocarrier, gene therapy, combination therapy, immunological response

## Abstract

Bacteriophages have emerged as versatile tools in the field of bioengineering, with enormous potential in tissue engineering, vaccine development, and immunotherapy. The genetic makeup of phages can be harnessed for the development of novel DNA vaccines and antigen display systems, as they can provide a highly organized and repetitive presentation of antigens to immune cells. Bacteriophages have opened new possibilities for the targeting of specific molecular determinants of cancer cells. Phages can be used as anticancer agents and carriers of imaging molecules and therapeutics. In this review, we explored the role of bacteriophages and bacteriophage engineering in targeted cancer therapy. The question of how the engineered bacteriophages can interact with the biological and immunological systems is emphasized to comprehend the underlying mechanism of phage use in cancer immunotherapy. The effectiveness of phage display technology in identifying high-affinity ligands for substrates, such as cancer cells and tumor-associated molecules, and the emerging field of phage engineering and its potential in the development of effective cancer treatments are discussed. We also highlight phage usage in clinical trials as well as the related patents. This review provides a new insight into engineered phage-based cancer vaccines.

## 1. Introduction

Cancer is a complex disease involving the irregular and uncontrolled growth of cells, making it difficult to treat [1]. The incidence of cancer is increasing; however, owing to the diversity of tumors and their underlying causes, comprehensive therapy for each patient has been difficult to develop [1]. Surgical resection, chemotherapy, and radiation therapy have been the mainstay of cancer treatment for decades [2]. Though surgery is the preferred method for the eradication of primary tumors, in certain cases, not all cancer cells are eradicated, resulting in tumor recurrence. Treatments based on chemotherapy or radiation therapy have the potential to induce considerable cytotoxicity in normal cells, which excludes them as therapeutic options for certain cancer types or patients [3,4,5].

Conventional therapies are unable to selectively eliminate cancer cells while simultaneously conserving normal cells and tissues. Several factors must be considered when developing cancer-targeting medications, including the challenging microenvironment in which cancer cells thrive. Cancer research is driven by the goal to develop an effective therapeutic strategy based on novel treatment and prevention methods [2]. Considering the impact of immunology, the tumor microenvironment is a key aspect of this new therapeutic paradigm. Moreover, the number of available treatments has increased because of advances in gene editing and other biotechnological techniques [6]. Cancer treatment has transitioned from conventional techniques to modern approaches, including targeted therapy and immunotherapy [2,7,8] where virus-based cancer therapy has been recently proposed.

**Figure 1:**
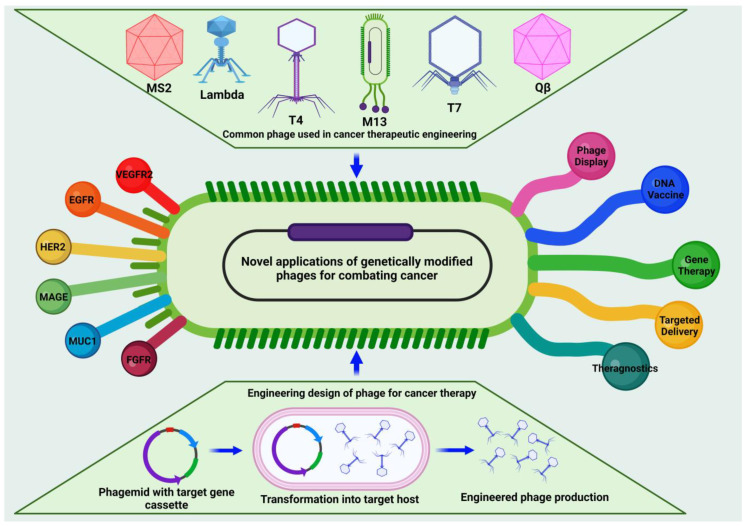
Schematic presenting the engineering designs, applications, and types of phages used in cancer immunotherapy.

Many different types of viruses, both mammalian and bacterial, have been used for several types of biological applications [9,10,11,12,13,14,15,16,17,18,19,20,21,22,23,24,25,26]. Bacteriophages, i.e., prokaryotic viruses that exclusively infect bacteria, not mammalian cells, have recently attracted the attention of the scientific community as potential cancer immunotherapy agents. This is due to the ease with which they display the desired functional peptides on the body in order to achieve high specificity towards cancer cells without incurring unexpected immunogenic issues in humans. Bacteriophages exist in a variety of forms, and each form has a unique structure and is associated with a unique strain of bacteria. Bacteriophages have several potential uses, including in the phage display technique and as phage vectors for gene therapy [12,13,14,15,16,17,18,19,20,21,22,23,24,25,26,27,28,29,30,31]. Phages capitalize on genetic plasticity to alter their surface, thus opening a new dimension in the prospective use of bacteriophages for medicinal purposes. Protein–protein interactions, receptor-binding sites, epitopes, mimotopes, and functional sites of antigens may be quickly and efficiently detected using the phage display approach [32]. Figure 1 provides a comprehensive summary of the various types, designs, and applications of engineered phages described in this review and outlines the different approaches used to engineer phages and the diverse applications of these methods, serving as a valuable visual aid in the understanding of the key concepts presented in this review.

Bacteriophages (phages) can accommodate large transgene inserts and can amplify and purify these gene inserts in a cost-effective manner, making them effective potential candidates for gene delivery [33]. Moreover, they have the potential for use in cancer treatments because they can selectively target certain cellular and tissue components. Selective phage-mediated cancer therapeutics involve the interaction of viral genetic material with cellular molecular mechanisms. Bacteriophages are used to combat cancer; therefore, understanding how mammalian cells react to phages in vivo is essential [34]. As potential in vivo agents, the biodistribution and effects of bacteriophages on normal tissues are important considerations that should be studied carefully before advancing these reagents into the clinic in large-scale trials.

In this review, we present the basics, rationale, and design of bacteriophages used in cancer immunotherapy, including their interactions with the immune system and other biological systems. The potential applications of phage therapy in combating cancer, along with specific topics including phage display systems, phage DNA vaccines, and combination therapy are addressed. The advantages and limitations of phage application are highlighted by the clinical trials and patents. We also discuss the current trends and prospects of bacteriophage research in cancer immunology.

## 2. Bacteriophages: Exploring the Intricacies of Their Structure and Biology

### 2.1. The Structure and Evolution of Bacteriophage

Bacteriophages have considerable potential as therapeutic alternatives for molecular biology-based treatment methods because of their nanoscale, polyvalent surface characteristics, and nonpathogenic nature. They can infect almost every known bacterial species and have played an important role in the evolution of bacteria and other prokaryotes. The growth of phages relies on the genomic and proteomic components of the host. Phages are abundantly distributed wherever bacteria thrive, and more than 10^30^ phage particles are estimated to be present in a wild-type bacterial strain. Frederick W. Twort of Britain (1915) [35] and Félix d’Hérelle of France (1917) [36] independently discovered bacteriophages, and d’Hérelle coined the term “bacteriophage”, which derived from the term “bacteria” and the Greek term “phagein”—which means to devour—owing to its bactericidal activity. After the discovery of phages, several researchers, including d’Herelle, considered them for the treatment of pathogenic bacterial infections and diseases such as *Staphylococcus aureus meningitis* [37], dysentery [38], and suppurative illnesses [39]; however, interest in the use of bacteriophages for treating bacterial infections waned after the discovery of penicillin by Alexander Fleming in 1928.

The intractable challenge of antibiotic resistance and our extensive understanding of phage biology have sparked considerable interest in phage therapy, particularly phage vaccines. Antigenic peptides displayed by whole filamentous phages were originally employed as a vaccine against *Plasmodium falciparum* in a rabbit model in 1988 [40]; thereafter, several studies have investigated phages as potential new vaccine candidates [41,42]. Scientists from several fields, including those concerned with protein–protein and protein–ligand interactions, medical diagnostics, gene and drug delivery vehicles, and biomedical engineering, have extensively investigated phages [12,13,14,15,16,17,18,19,20,21,22,23,24,25,26,41,42,43,44,45,46].

Bacteriophages are being extensively investigated owing to their well-defined three-dimensional structure and genomic architecture. The head of a phage consists of single- or double-stranded DNA or RNA surrounded by a capsid [47]. The attachment of bacteriophages to bacteria is facilitated by receptor-binding proteins present in their tail fibrils or capsid surface, which bind to cell surface receptors, including glycoproteins, lipopolysaccharides, amino acids, teichoic acids, or pili fragments, through electrostatic forces, resulting in irreversible contact [48,49,50]. Once a phage binds irreversibly to a bacterium, the viral tail protein coat is compressed, and the stiff tail tube penetrates the cell membrane. After penetrating the periplasm, the tube injects the viral genome into the bacterial cytoplasm and uses enzymes, mostly lysozymes, to degrade the peptidoglycan in the bacterial cell wall [51]. For example, filamentous bacteriophages use cell pili to invade bacteria by binding to phage minor pIII proteins. Upon binding to the bacterial pilus, the phage induces its retraction, which facilitates the phage’s entry into the cell. Once inside, the phage attaches to the TolA protein within the TolQRA complex, leading to the exposure of the pIII protein in the periplasm and subsequent injection of viral DNA [52]. After binding to a bacterium, the phage introduces its genetic information into the bacterial cell in a lytic or lysogenic manner. The lytic cycle involves the invasion of the host cell, replication of the phage inside the host, and finally the destruction of the host cell to release more phage particles. The lysogenic cycle involves the insertion of phage nucleic acids into the host chromosome without killing the cells. Other cycles, such as pseudo lysogeny and chronic infections, have also been reported [53]. As a part of the lytic cycle, the cell metabolism of the host is restructured to facilitate phage replication, transcription, and translation. The lysogenic cycle involves the replication of both the phage genome and the host at the same time. Most lysogenic cycles persist until the transition to the lytic cycle [54].

### 2.2. Phages in the Human Body and Their Pharmacokinetics

Phages are also present in the human body, where they feed on microbiomes in the skin, oral cavity, lungs, intestines, and urine system, and control bacterial population dynamics [55]. They comprise the majority of the gut microbiome in humans and may enter the body through the leaky gut or via a Trojan horse approach [56]. Phages can readily transcytose human tissues and are often detected in human blood, despite the absence of mammalian/human cell tropism [57]. Moreover, phages can easily pass through cell membranes; approximately 31 billion phages enter the body every day through the digestive system [58].

Bacteriophages actually have considerable potential as therapeutic alternatives for molecular biology-based treatment methods because of their nanoscale, polyvalent surface characteristics, and nonpathogenic nature. They are under extensive investigation owing to their well-defined three-dimensional structure and genomic architecture. Phages range in size from 40 to 200 nm, with the exception of filamentous phages, which are often less than a few micrometers in size [59]. They contain well-defined and repetitive structural units with minimal surface variations, mostly composed of conserved proteins and simple nucleocapsids of nucleic acids and proteins, this results in a predictable and comparable immunological response across closely related phages [60,61]. However, their size can affect their processing and clearance by the reticuloendothelial system (RES) or hepatobiliary system.

Phages can effectively reach tumors owing to their ability to cross-leak blood vessels; however, they cannot cross-leak normal vessels, limiting their off-site diffusion. The RES is responsible for phage clearance, and, post clearance, they are processed for elimination by splenic or liver macrophages. Some phage variants, known as long-circulating phages, have a longer blood half-life because of single capsid mutations that circumvent the RES clearance process and prolong circulation time. Therefore, phages can be used as targeted delivery vectors for cancer treatment. However, the passage of biological barriers by phages requires further exploration of barrier biology to enhance phage efficacy. The surface charge of phages affects in vivo pharmacokinetics, and negative charges of −30 to −10 mV at physiological pH may result in good blood circulation [62,63]. Phage efficacy can be improved with neutral or slightly negative charges and PEGylation to increase their half-life and avoid clearance by the RES system. Phages are stable over a wide range of pH and temperature conditions and can remain stable for 24 h at pH 3–11 [64]. They are also stable through freeze-drying, desiccation, and freeze–thaw cycles and at 63 °C for up to six weeks [65,66]. Recent studies have shown that buffered infusion solutions, often used in the medical field, are ideal for maintaining phage stability [67].

## 3. Exploring Phage–Immune Cell Interactions and Administration Modalities

Engineered bacteriophages displaying specific molecules on their surface have a significant advantage over traditional cancer therapies because of their ability to be highly specific in targeting of cancer cells without affecting healthy cells. Phages can be engineered to carry therapeutic molecules or antigens that can boost their ability to trigger an immune response against cancer [68,69,70]. An effective approach involves displaying tumor-specific antigens on the phage surface, which can be recognized and destroyed by immune cells. Animal studies have elucidated the way that phage-based vaccines can generate potent and durable antitumor immune responses [71,72,73]. In addition to their efficacy, phages are safe, cost-effective, and easy to produce, making them a feasible option for large-scale vaccine development and distribution [74,75]. Thus, phages have introduced a new technique for the immunotherapy of diverse types of cancer, resulting in better outcomes for patients and a potential cure for cancer.

**Figure 2:**
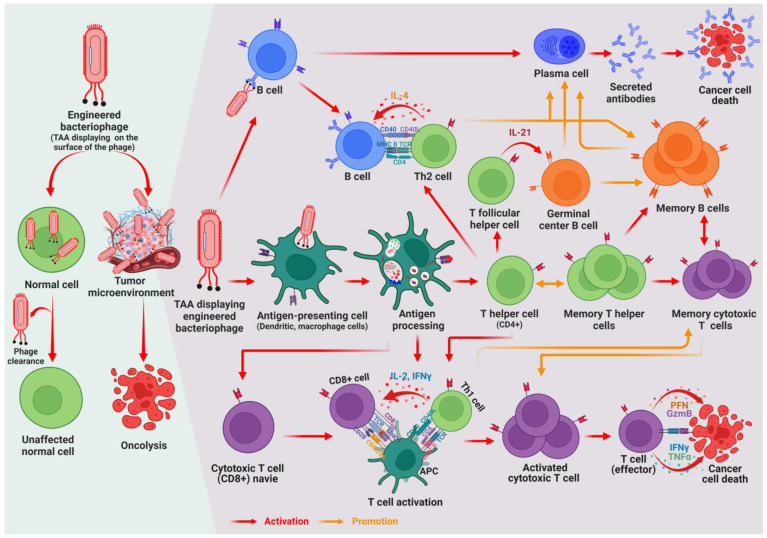
Schematic illustration of the impact of engineered phages on both normal and cancer cells. Phages do not affect normal cells as they do not exhibit innate mammalian or human cell tropism. However, when a phage is engineered to display the cancer antigen on its surface, it triggers an immune response that destroys cancer cells to treat the tumor. Upon encountering a phage with surface-displayed antigens, antigen-presenting cells (APCs) phagocytose and decompose the phage. The APCs fragment the antigen into smaller peptides, which are then presented on their surface by major histocompatibility complex class II (MHC-II) molecules. The presented antigens activate various types of cells, including CD4+ T helper cells, B cells, and CD8+ cells. B cells differentiate into plasma cells that produce antibodies, which destroy cancer cells. Meanwhile, T helper cells differentiate into subtypes with specific functions, such as Th2 cells that help mature humoral responses and T follicular helper cells (Tfh), which are important for the maturation of memory B cells and high-affinity antibody-producing plasma cells with long life. Another subset of CD4+ T cells become memory T helper cells. Th1 T helper cells play a crucial role in forming cellular responses by interacting with APCs to activate CD8+ cells or cytotoxic T lymphocytes (CTLs), which cause the apoptosis of cancer cells. Memory cytotoxic T cells also restore the CTL response by contact with secondary antigens.

The interaction between phages and immune cells is an important factor in evaluating the potential of phage-based vaccines (Figure 2). Two primary aspects of this interaction have been identified: phage immunogenicity and immunomodulatory activity. Phage immunogenicity refers to the ability of phages to trigger specific immune responses, such as the production of antibodies against phage antigens [68]. Phages can induce cellular responses that are essential for the inhibition of viral infection and the clearing of tumor cells. They also face challenges related to the generation of anti-phage antibodies and phage–antibody interactions, which can lead to the inactivation of phages and can limit the effectiveness of phage-based vaccines. In contrast, phage immunomodulatory activity refers to the nonspecific effects of phages on the different populations of immune cells involved in both innate and adaptive immune responses [68].

Phages have the ability to engage with pattern recognition receptors (PRRs), notably toll-like receptors (TLRs), which identify pathogen-associated molecular patterns (PAMPs) and damage-associated molecular patterns (DAMPs). The engagement between phages with PRRs activates innate immunity pathways, cytokine generation, and immune cell recruitment to infection sites, leading to the establishment of an adaptive immune response commenced by antigen-presenting cells (APCs) such as dendritic cells (DCs). Phages engulfed by APCs and a foreign antigen expressed on an engineered phage can be presented via major histocompatibility complexes (MHCs), recognized by naive T cells, and activated toward effector or memory functions. The antigen presentation on MHC-I leads to the differentiation of CD8+ T cells into cytotoxic T lymphocytes (CTL) that directly kill infected host cells, while antigen presentation on MHC-II triggers CD4+ T cell differentiation into T helper (Th) cells that assist B cells to produce specific antibodies against antigens, provided that the APC also displays costimulatory ligands [69,70,76]. Therefore, the design rationale and use of phage-based vaccines require comprehensive knowledge of the interactions between phages and immune cells. Considering both phage immunogenicity and immunomodulatory activity, researchers can develop more effective vaccines that stimulate humoral and cellular responses.

Subcutaneous and intramuscular injections are the most common routes for phage vaccine administration. However, these methods can be invasive and uncomfortable and require trained medical personnel for administration. Therefore, alternative routes of vaccine delivery, such as oral administration, are being developed. Consequently, M13 phage particles have emerged as potential candidates for oral vaccines because they can cross the gastrointestinal barrier and remain stable [77,78]. However, potential harm to the host gut bacteria is a reason for concern, as phages are known for their ability to recognize and infect bacteria [79]. A double-blinded, placebo-controlled crossover trial examined the effects of a cocktail of *Escherichia coli*-targeting bacteriophages on gut microbiota and inflammatory markers in a healthy human population. Results show that the phages selectively reduced target organisms without globally disrupting the gut community, demonstrating their potential for a targeted approach to the modification of the gut microbiota [80]. Recent study on the phage vB_KpnP_K1-ULIP33 involved its evaluation in an in vitro model of the human intestinal microbial ecosystem, namely the Simulator of the Human Intestinal Microbial Ecosystem (SHIME^®^) system. The results show no significant impact on the global colonic microbiota, suggesting that further studies are needed to assess its efficacy against its bacterial host in the human intestinal ecosystem [81].

Various approaches have been developed to ensure that phage-based vaccines are safe for oral administration. One strategy is to use non-lytic phages such as M13 that do not destroy bacterial cells, thus reducing the risk of damaging the gut microbiome of the host, whereas another strategy is to use viral particles with non-functional tail fibers, required for target recognition and further infection [32]. These viral particles can act as vaccine delivery systems without infecting bacterial cells, thereby minimizing the risk of harm to the host. Despite concerns, the use of phage-based vaccines for oral administration has demonstrated strong immunostimulatory effects and has led to the development of new strategies for enhancing the safety of phage-based vaccines, reinforcing the use of phage particles as oral vaccine carriers [82,83].

## 4. Phage Engineering: Innovative Approaches for Design and Development

Phages have promising applications in vaccine development, drug delivery, and cancer targeting [43,79,84]. Phage DNA vaccines utilize phages to deliver protective antigens or mimic epitopes in the form of DNA vaccines [65]. Phage-displayed vaccines use recombinant phages displaying immunogenic peptides or proteins on their surfaces, which can be customized with desired affinity properties. Biopanning is an effective selection technique that enriches a diverse phage library by immobilizing it on a receptor on a solid support, washing away unbound phages, and resulting in an enriched phage population with high affinity for the specific receptor [85]. This technique is used to isolate peptides that bind to a specific target molecule based on their binding affinities [10,85,86]. Biopanning is categorized into in vitro and in vivo biopanning, with the former used for selecting high-affinity peptides for biomolecules or inorganic materials [44,87,88] and the latter for obtaining cell/tissue-targeting peptides [89,90].

Phages can be used to target cancer cells and drug delivery by constructing diverse self-assembled nanostructures for the diagnosis and treatment of diseases. In vitro selection using immobilized cells as targets is used to achieve this specificity, and the biopanning method is used to select phages [85,91]. Ff class filamentous phages are commonly used as phage display vectors due to their unique composition, and modifying major coat proteins can enable specific targeting of certain cell types, particularly for cancer [92,93]. In vivo biopanning involves the injection of a random phage-displayed peptide library into animals to circulate and bind to the target cells or tissues, which is useful for isolating target-binding affinity peptides.

Phages are also effective in targeting specific tissues and organs for drug delivery or imaging, including the brain, retina, skin, pancreas, lungs, adrenal glands, uterus, and intestines [10,90,94]. Targeting specific tissues and organs can be challenging because of the relative impermeability of the blood–brain barrier (BBB). Li et al. used phage display combined with in vivo screening to isolate a brain-targeting peptide (Pep TGN), which was conjugated onto nanoparticles, and it enhanced brain accumulation efficiency in mice [90]. Moreover, many tissue-targeting peptides have been discovered, including motifs for various cancers, including leukemia; head and neck, lung, breast, cervical, ovarian, liver, melanoma, gastric, colon, prostate, and esophageal cancers; lymphoma; and osteosarcoma [85]. Examples of both pre-clinical and clinical studies of bacteriophage, as well as peptides that have been identified or engineered for cancer therapy using phages, are discussed later in this article.

Phage display technology provides two methods for enhancing vaccines: direct display, in which a foreign gene sequence is fused with a coat protein; and indirect display, which involves an antigen-binding peptide [95]. Direct display is cost-effective and efficient but may restrict antigen diversity and conformation. Conversely, indirect display enhances antigen diversity but is more complex to manufacture. Filamentous phages, T4 phages, T7 phages, phage lambda, and RNA phages are phage display systems commonly employed for vaccine development and delivery, some of which are discussed in this review. Table 1 provides the distinct features of commonly employed bacteriophage strains in phage display studies, encompassing their dimensions, genomes, coat proteins, and copy numbers.

### 4.1. Filamentous Phage Display Systems

Filamentous phages, such as M13, fd, and f1, infect gram-negative bacteria, making them ideal for creating phage-displayed vaccines with unique structure of coat proteins and single-stranded DNA genomes assembled into nanofibers. Filamentous phages can be continuously produced and secreted by the host bacterium without killing it, making them useful for the rapid development of vaccines against emerging threats. The filamentous phage display system is one of the most efficient display systems because all five coat proteins can display antigens. Engineering of the pIII minor coat protein is well known for its ability to insert foreign peptides. A phage display system has been developed as an information mining tool for the identification of peptides that mimic functional peptides and the study or targeting of the roles of chemokines and receptors [96,97,98,99]. pVIII has been used in tissue engineering and cancer immunotherapy [74]. Foreign peptides displayed on pIII have been used to fabricate multifunctional synthetic phages for sensing or capture [20,22,23,100,101]. Additionally, landscape peptide presentation on the major coat protein has been utilized to develop a template for inorganic crystals used in energy and memory storage devices and to make stimulus-responsive materials [102,103,104,105,106]. Phages have also been used for medical applications, such as targeted drug delivery, gene delivery, imaging agents, sensors, and tissue engineering scaffolding materials [12,13,14,15,16,17,18,19,20,21,22,23,24,25,26,100,101,107,108,109,110,111,112]. For example, Wang et al. have developed a novel therapeutic platform using the M13 phage to display the antitumor cytokine granulocyte-macrophage colony-stimulating factor (GM-CSF), a potent activator of STAT5 signaling in murine macrophages. The GM-CSF phage caused a significant reduction in tumor size and increased the number of CD4+ lymphocytes compared with that caused by the unmodified phage in a murine colorectal cancer model. The GM-CSF phage therapy in combination with radiation therapy results in a 100% survival rate and a 25% complete remission rate [113]. In another study, an M13 phage was engineered to selectively bind to different types of collagen using collagen-mimetic peptide motifs. The engineered phage efficiently detected and bound to abnormal collagen and could be conveniently attached to fluorescence imaging agents to monitor and diagnose various pathological conditions, including cancer [114].

### 4.2. T4 Phage Display Systems

The T4 bacteriophage has a complex structure consisting of a head, a tail, and 12 tail fibers, with three major proteins: gp23, gp24, and gp20, and two non-essential coat proteins: a highly outer capsid protein (HOC) and a small outer capsid protein (SOC) [115]. During infection, dsDNA is released through gp20, forming a portal to infect *Escherichia coli*, whereas the SOC protein protects the phage head from pH, heat, and enzymes [116]. The SOC protein allows the display of a foreign protein of interest at the N-terminus, whereas the HOC protein allows display at the C-terminus. [117]. The study revealed that the role of SOC in stabilizing the T4 phage capsid protein enables the fusion of a mutated F1 protein with V antigen, creating a potent vaccine against *Yersinia pestis* that provides full protection in rodent and cynomolgus macaque models through the use of recombinant T4 phage [118]. T4 phage display systems can be used both in vivo and in vitro, with an in vitro approach capable of displaying large and complex proteins on the phage surface [119,120]. One of the most significant features of T4 phages is their ability to display foreign antigens on the HOC and SOC proteins, which are highly immunogenic [121]. T4 phage is a potential vaccine vector candidate because of its ability to package multiple pathogenic genes encoding various vaccine antigens. The stability provided to the phage capsid by the SOC protein makes it an excellent option for vaccine administration and transport. PC-3 tumor cells exposed to T4 and M13 bacteriophages have exhibited significant upregulation of integrin genes and reduced migration activity, suggesting the potential of bacteriophages in anticancer therapies through their ability to trigger internal cellular signaling, gene expression alterations, and broad binding properties for drug delivery and therapy [122].

### 4.3. T7 Phage Display Systems

The T7 phage has a robust icosahedral capsid enclosing a 40-kb dsDNA genome assembled with 415 capsid proteins. It has two major capsid proteins covering the head and tail, with gp15 and gp16 assisting in DNA insertion. Non-essential gp10B can be used for antigen display, with antigen density affecting the immune response. T7 phages are a convenient display system for proteins that have difficulty crossing membranes or that prefer a reducing environment for folding [123]. However, the copy number of the displayed peptides or proteins may decrease as the sequence size increases. The T7 phage has several advantages over other phages, such as rapid proliferation and tolerance to foreign gene insertions of up to 2 kb without affecting structural integrity, making it a cost-effective and versatile vector for display systems [124]. The Tat protein can be displayed on the surface of T7 phages to improve delivery efficiency, making it suitable for DNA vaccine delivery [125,126]. Copper ions can be loaded onto T7 bacteriophages to target cancer cells via ligand-mediated transmembrane transport. The copper-loaded T7 phage displays a single peptide that targets cancer cells via aV*β*3 integrin and maintains its stability and structure even under harsh conditions. The intake rate of the copper-hybridized T7 phage particles by MCF-7 cancer cells is 1000 times higher than that of the control group [127].

### 4.4. Phage Lambda Display Systems

Phage lambda, a member of the family Siphoviridae, has an isometric head and a flexible tail made up of 405 copies of the head protein D and 32 disks, each consisting of six copies of the major tail protein pV. The lambda phage can display multiple copies of complicated large proteins, making it an effective vaccine delivery vehicle [128,129,130,131,132,133,134], and has a 48.5-kb long linear dsDNA genome that exhibits both lysogenic and lytic lifecycles. In the lysogenic state, the lambda genome replicates with the bacterial genome, while the lytic stage requires the excision of the lambda genome into an extrachromosomal circular DNA template and the production of phage proteins [135]. A lambda bacteriophage nanoparticle-based vaccine was developed to induce robust cytotoxic T lymphocyte activity against human epidermal growth factor receptor 2 (HER2)/neu-overexpressing tumors in a BALB/c mouse xenograft tumor model [136]. A nanoparticle system was recently designed using phage-like particles, which can target specific cancer cells for the intracellular delivery of therapeutics and imaging. This system can be tailored to offer a platform with diverse yet specific functionalities for cancer treatment [137].

### 4.5. MS2 Phage Display Systems

The MS2 phage, a member of the Leviviridae family, is one of the smallest and simplest phages, with a 26-nm icosahedral size. Its 3.57-kb long single-stranded RNA genome encodes the major coat protein CP, maturation protein A, replicase, and lysis protein L. The small size of MS2 may enable it to quickly transport antigens to immune tissues to encounter immune cells [138]. MS2 phages employ the same method as that used by the filamentous phages to introduce their genome into *E. coli* by attaching it to the F pilus [92]. However, MS2 phages differ from filamentous phages in that they require host cell lysis to release new viral particles. MS2 phages also affect the expression of genes associated with prostate cancer cell survival and proliferation by reducing/impairing prostate cancer cell viability via caveolin-mediated endocytosis [139].

### 4.6. Qβ Phage Display Systems

Qβ phage, a small RNA virus in the Leviviridae family, encodes proteins including the major coat protein CP, maturation protein A2, and replicase in its 4.2 kb genome. It infects host cells using the F pilus of E. coli, similar to the MS2 phage, and the A2 protein can cause lysis by inhibiting cell wall synthesis [140]. Qβ phages can also express the A1 protein, which displays antigens through its β-hairpin in the read-through domain consisting of 196 amino acids [141,142]. The display of tumor-specific antigens on the surface of engineered Qβ phages can be used for inducing an immune response against cancer cells. The Qβ virus-like particles (VLPs) have been used to display weak Tn antigens as potential targets for anticancer vaccines. The display of Tn antigen on Qβ phages elicits strong humoral responses and high Tn-specific IgG titers. Qβ phages present a highly attractive platform for the development of carbohydrate-based anticancer vaccines owing to their ability to display antigens in an organized and high-density manner [143]. However, the use of the Qβ phage in cancer treatment is still in the early stages of research, and further studies are needed to determine its efficacy and safety.

## 5. Revolutionary Strategy of Engineered Bacteriophages for Combating Cancer

### 5.1. Role of Phages as Antigen Display Systems for Vaccines

Phage display is a powerful tool for the development of vaccines and identification of new protective antigens [79,84]. This technology can be used for two main processes: antigen display on phage vectors and identification of novel antigens [43]. Phage display technology offers an effective approach to the identification of cancer cell surface markers and the development of functional anticancer peptides for therapeutic use. By conjugating antigens to phage surface proteins, phage display vaccines can generate tailored immunogenic viral particles [32]. Several candidates have been evaluated as phage-based cancer vaccines in pre-clinical research studies, including epitopes from the vascular endothelial growth factor receptor 2 (VEGFR2), epidermal growth factor receptor (EGFR), HER2, melanoma antigen gene (MAGE), mucin 1 (MUC1), fibroblast growth factor receptor (FGFR), Fms-like tyrosine kinase 4 (Flt4), and mimotopes of tumor-associated carbohydrate antigens (TAA). Some of these anticancer phage vaccines have been successfully used in cancer immunotherapy [42,144,145,146,147,148,149]. Table 2 summarizes the peptides identified or engineered using phages for cancer therapy.

#### 5.1.1. VEGFR2 Antigen Display

VEGFR2 is a potential immunotherapy target for different types of malignancies. VEGFR2, also known as kinase insert domain receptor, is a transmembrane receptor protein that plays a crucial role in the regulation of angiogenesis (formation of new blood vessels) and lymphangiogenesis (formation of new lymphatic vessels). It belongs to the family of receptor tyrosine kinases (RTKs) and is primarily expressed on the surface of endothelial cells that line the interior surfaces of blood vessels. Phage display technology has been used to select peptides that can act as antagonists to inhibit the activity of VEGFR2 and suppress VEGF-mediated angiogenesis [150,163,164]. Self-antigens in tumors are often overexpressed and, therefore, not well-recognized by the immune system; however, immunotherapy that targets them have been reported to be effective [144,165]. VEGFR2 is overexpressed in tumor endothelial cells and can be used as a tumor-associated antigen. A VEGFR2-based vaccine was developed using a phage display system, which could generate anti-VEGFR2 antibodies that inhibited tumor growth in mice through CD4+ T lymphocytes. The use of T4 phages as a vaccine carrier may help overcome immune tolerance to VEGFR2 [144]. Administration of VEGFR2 extracellular domain-expressing T4 recombinant phages inhibits VEGF-mediated tumor angiogenesis by binding specifically to vascular endothelial growth factor (VEGF), thus inhibiting downstream signaling pathways and suppressing tumor growth and microvascular density in vivo [166]. Monoclonal antibodies, such as bevacizumab (avastin), and peptides selected using a phage display system have been developed to counteract VEGF-dependent tumor angiogenesis [167,168,169,170,171]. The ATWLPPR (A7R) peptide is a potential inhibitory agent for cancer-associated angiogenesis and metastasis and has been explored for additional biomedical applications, such as the imaging of tumor vasculature and targeted drug delivery [150,172]. Other peptides, such as CPQPRPL, K237-(HTMYYHHYQHHL), and F56, have been selected using phage display systems to block the binding of VEGF to its receptors and inhibit tumor growth and metastasis. These pan-VEGF inhibitory peptides may be powerful anti-angiogenic drugs because they target all three members of the VEGFR family [152,164,173].

#### 5.1.2. EGFR Antigen Display

EGFR is a transmembrane protein that plays a crucial role in regulating epithelial tissue development [174,175,176]. EGFR is overexpressed in several types of cancers and is considered a potential target for cancer therapy. Targeted therapies, such as monoclonal antibodies and tyrosine kinase inhibitors, have been developed; however, acquired drug resistance is a common problem. Studies have examined the use of EGFR peptide ligands as promising tools for the targeting of overexpressed EGFR receptors in different cancer cell types [177]. Phage display technology has been used to identify high-affinity peptides and antibodies that can bind to EGFR and inhibit its signaling pathway, leading to the inhibition of tumor cell proliferation and survival. These phage-derived ligands have exhibited potential in the development of targeted therapies for EGFR-positive cancers [42,146]. Another study elucidated the way in which antagonistic anti-EGFR nanobodies that were selected using the phage display technology effectively blocked the binding of endothelial growth factor (EGF) to EGFR and EGF-mediated signaling and delayed tumor outgrowth in vivo [178]. Phage display screening with panitumumab-isolated EGFR mimotopes (P19 and P26) and HSP70-P19/P26 fusion proteins reduced tumor growth in lung cancer models, indicating their potential for anti-EGFR immunotherapy [151].

#### 5.1.3. HER2 Antigen Display

Human epidermal growth factor receptor 2 (HER2) is a transmembrane receptor belonging to the group of EGFRs. It plays a crucial role in regulating cell growth and division, and its overexpression is associated with the development and progression of several types of cancers, including breast, gastric, and ovarian cancers. HER2 is a potential therapeutic target for HER2-positive breast cancer. Monoclonal antibodies that specifically bind to and block the activity of HER2, such as trastuzumab and pertuzumab, have been developed and are currently used to treat HER2-positive breast cancers. Studies have focused on vaccines for cancer cells expressing HER2 in an attempt to elicit an immune response and, eventually, destroy these cells. The HER2-derived peptides E75 and AE37 have been investigated for their ability to stimulate CTLs to recognize and kill HER2+ cancer cells [145,153]. Phage-based vaccines were engineered using these peptides and tested in vivo in BALB/c mice implanted with TUBO cells. The λF7-gpD::E75 vaccine exhibited a delay in tumor growth [153], whereas λF7-gpD::AE37 was shown to be effective as both preventive and curative effects were observed [145]. Phage display technology has also been employed to identify specific tumor-associated regions of the HER-1 and HER-2 proteins [179]. This technology can also produce single-domain antibodies (sdAbs) that target the HER2-tyrosine kinase in HER2-positive breast cancer, exhibiting potential as a therapeutic option by inhibiting kinase activity and reducing cell viability [180]. Moreover, phage-based HER2 vaccines delay the onset of mammary tumors, reduce tumor growth and multiplicity, and produce anti-HER2 antibodies that decrease cell viability, providing a promising alternative strategy for the treatment of HER2-positive breast cancer using both trastuzumab-sensitive and trastuzumab-resistant BT-474 cells [181].

#### 5.1.4. MAGE Antigen Display

MAGE is a group of tumor-associated antigens that are frequently overexpressed in several types of human cancers. The identification and targeting of MAGE is being actively explored for cancer immunotherapy. The MAGE family includes several X-linked genes that encode tumor antigens recognized by CTLs. MAGE proteins are found in human cancer cells but not in normal tissues, making them valuable TAAs. Phage display technology has been used to select MAGE-specific antibodies that can recognize and potentially target MAGE-expressing tumor cells. In vitro and in vivo experiments have revealed that phage vaccines containing MAGE-A1_161–169_ phages induced immune responses, controlled tumor growth, and prolonged overall survival [147]. Similarly, studies have shown that fd23/Mg10 and fd23/Mg3 phages induce cytotoxic responses in T lymphocytes and splenocytes and reduce tumor growth in HHD transgenic mice [155]. Additionally, oral immunization using T7 phages displaying TAAs, such as MAGE-1-T7, SSX2-T7, p53-T7, and Hsp27-T7, induces effective Th1 and Th2 responses against antigens [182]. Immunization using pC89hisP1A phages has resulted in the generation of Th1-dominated immune responses and has prolonged the survival of mice challenged with P815F3 cells [183]. Next-generation phage-display mimotope variation analysis has identified antibody responses to targeted cancer therapies, revealing melanoma-associated and cancer-testis antigens linked to DC vaccines and anti-PD-1 monoclonal antibodies [184].

#### 5.1.5. MUC1 Antigen Display

Overexpression of the transmembrane glycoprotein MUC1 is also observed in many types of cancers. MUC1 comprises a heavily glycosylated extracellular domain and a transmembrane domain that anchors the protein to the cell membrane. A study has demonstrated that Qβ phages could produce VLPs conjugated with MUC1 peptides. VLPs induce the production of specific antibodies and CTL responses in mice, which could bind to and kill MUC1-expressing tumor cells through complement-mediated cytotoxicity [148]. The Variable Epitope Library (VEL) vaccine platform was used to target the overexpressed MUC1 antigen in tumors. Two murine MUC1-derived epitopes were used to generate VELs, which reduced tumor area and lung metastasis. Additionally, MUC1 variable number tandem repeat (VNTR)-based VELs reduced metastatic burden when used with DCs and M13 recombinant bacteriophages [185].

#### 5.1.6. FGFR Antigen Display

The FGFR family plays an important role in regulating various physiological and pathological processes, such as epithelial-mesenchymal interactions, embryonic development, metabolism regulation, migration, and angiogenesis [186]. FGFR mutations are frequently associated with genetic disorders and various types of cancers [187,188,189,190,191,192,193,194]. Therefore, the development of specific peptide ligands that target overexpressed FGFR is a strategic approach for cancer therapy. Phage display technology has been used to identify specific peptide ligands of different FGFR isoforms. For example, the LSPPRYP peptide was identified by screening a phage display library using murine fibroblasts overexpressing FGFR1c and FGFR2c isoforms and human keratinocytes overexpressing FGFR2. This peptide exhibited strong antiproliferative activity in vitro and in vivo and competed with the natural ligand bFGF for binding to its receptor [156]. Similarly, the peptide VYMSPF was identified by screening a library of 6-mer phage peptides using Sf9 insect cells to select specific peptide ligands of FGFR1. This peptide inhibits the mitogenic activity of aFGF, thus exhibiting antagonistic activity against aFGF for the treatment of human angiogenic diseases [157]. Other peptides have been identified using similar phage display techniques and have shown promising results in inhibiting cell proliferation and migration in different cancers, such as breast and ovarian cancers. For example, the P7 peptide was identified by screening a library of random heptapeptides in bFGF-stimulated murine fibroblasts [195]. This peptide inhibits cell proliferation by acting on the cell cycle and the MAPK signaling pathway and has shown potential for targeted therapy of breast and ovarian cancers [196,197,198]. The overexpression of FGFR1 in lung and breast cancers has led to the development of drugs that target FGFR1 signaling and ligand binding. The cyclic peptides F8 (ACSLNHTVNC) and G10 (ACSAKTTSAC) were selected using a phage display library to bind to the fibroblast growth factor (FGF)1–FGFR1 interface. The cyclic peptide F8 effectively prevented FGF1–FGFR1 interactions and reduced FGF1-induced proliferation of FGFR1-expressing cancer cells [158]. The P12 peptide (HSQAAVP) was also found to be a potential therapeutic target in prostate cancer. P12 binds to FGF8b, a major isoform expressed in prostate cancer, and inhibits FGF8b-induced cell proliferation by arresting the cell cycle and blocking the activation of certain signaling pathways. P12 is homologous to a region of the high-affinity FGF8b receptor, suggesting it may be more effective in interrupting FGF8b binding than other identified peptides [159].

#### 5.1.7. Flt4 Antigen Display

Flt4, expressed mainly in lymphatic endothelial cells, crucially facilitates lymphangiogenesis through its transmembrane receptor activity. In a study, T4 phages were genetically engineered to express murine Flt4, known to participate in tumor lymphangiogenesis and contribute to tumor progression and metastasis. The therapeutic potential of engineered T4-mFlt4 was evaluated through prophylactic vaccination in a mouse model. This study revealed that the prophylactic vaccination led to the production of targeted antibodies, which effectively suppressed lymphangiogenesis and distant metastasis while having no impact on tumor progression [149].

#### 5.1.8. Exemplary Array of Additional TAA Mimotope Variants

Phage display technology can also be used to identify peptides that mimic the structural and antigenic properties of proteins, carbohydrates, or lipids. These mimotopes have been selected for their specific fit with antitumor-associated antigen antibodies as they are more efficient than natural epitopes. For instance, the EM.L2 peptide (QHYNIVNTQSRV) fused with the L2 extracellular domain of human EGFR was expressed in phages, and the phages were tested in a mouse model of lung carcinoma, resulting in the activation of humoral and cellular responses and inhibition of tumor growth [160,199]. Phage display panning was also performed in mice bearing melanoma tumors, and a specific ligand WDC-2 (displaying the TRTKLPRLHLQS peptide motif) was identified in B16-F10 cells, which exhibited complete regression of the established tumors and long-term survival [161]. In another study, phage display was used to identify mimotopes of gonadotropin-releasing hormones (GnRH) that stimulated the production of therapeutic antibodies. In mice, immunization with phages displaying the selected GnRH mimotope induced long-lasting humoral responses and decreased testosterone production, which indicate that they may have the potential to treat hormone-sensitive cancers [73].

### 5.2. Role of Phage Nanocarriers in Transforming Cancer Treatment

#### 5.2.1. DNA Vaccines of Phages

DNA vaccines are highly advantageous over protein or peptide vaccines due to their accurate antigen folding and lack of downstream processing needs [200,201,202]. However, large primate trials showed poor immunogenicity, leading to the use of adjuvants in human trials [203,204]. Delivery vehicles are also required to overcome the instability and distribution issues associated with naked DNA vaccines [205]. In contrast, novel nucleic acid-based vaccines have demonstrated promising results in clinical trials, especially during the COVID-19 pandemic [206]. Phage particles possess adjuvant properties and are a practical option for DNA delivery. In bacteriophage DNA vaccines, eukaryotic expression cassettes containing target-specific bacteriophage promoters are used to insert antigen-encoding genes [32,200]. Phage DNA vaccines are a promising alternative to naked DNA vaccines, offering several advantages, including the ability to insert large DNA antigens of up to 20 kb [202,207,208]. Lambda phages have been extensively studied as vectors for the delivery of DNA vaccines that encode proteins such as green fluorescent protein (GFP) and hepatitis B surface antigen (HBsAg) using cytomegalovirus (CMV) promoter-controlled reporter genes. Not only are they a cost-effective gene delivery option, but they also offer distinct advantages in delivering nucleic acid immunization and targeting antigen-presenting cells for enhanced immune responses [208,209].

Research has shown that lambda-ZAP E7 bacteriophage-mediated gene transfer can efficiently deliver and express therapeutic genes, resulting in potent anti-tumor effects in vaccinated mice [210]. However, DNA vaccines targeting Δ16HER2, a protein implicated in breast cancer aggressiveness and drug resistance, failed to elicit immune protection in mice due to tolerogenic mechanisms. This challenge was overcome by engineering bacteriophages with immunogenic epitopes of Δ16HER2, which triggered a protective anti-Δ16HER2 response, breaking immune tolerance. These findings support phage-based anti-HER2/Δ16HER2 vaccination as a safe and effective immunotherapy against HER2-positive breast cancers [41]. Compared with peptide vaccination, lambda phage-based genetic vaccination induced immunological responses with higher antibody titers, cytokine production, and epitope binding driven by a Th1 response [211]. Filamentous phages are also being explored as vaccine vectors, allowing for immunization with multiple epitopes or antigens through a single delivery method [212]. Additionally, phage DNA vaccines are safe, simple, and inexpensive, without carrying antibiotic-resistance genes, and can be administered in multiple doses. They are also stable and offer better protein folding, the adjuvant effect of phage particles, and the presence of lipopolysaccharides and lipids, making them more effective than standard DNA vaccines [200,202]. RNA-based phage vaccines have not yet been studied for cancer immunotherapy in the available literature. However, to suppress viral replication and develop antiviral drugs for myocarditis, a study utilized artificial microRNAs that were linked to folate-conjugated bacterial phage packaging RNA (pRNA) [213]. Therefore, phage DNA vaccines are a promising approach for the development of safe and effective vaccines against various diseases.

#### 5.2.2. Gene Therapy of Phages

Researchers have long focused on developing gene therapies for cancer treatment [214]. However, a major hurdle has been the effective delivery of therapeutic genes to target cells [215]. Non-viral methods, such as naked DNA injection, gene gun, and chemical approaches, have limited efficacy [216]. On the other hand, eukaryotic viruses, such as lentiviruses, adenoviruses, and adeno-associated viruses (AAVs), have higher transfection efficiency and stable gene expression [216,217]. Despite this advantage, their use is constrained by natural cell tropism, immunogenicity, and oncogenicity risk [33,218,219]. Bacteriophages are a promising tool for cancer gene therapy due to their low cost and easy modifiability, despite initial limitations in transducing higher organisms [33]. An AAV transgenic cassette containing the herpes simplex virus thymidine kinase (HSVtk) gene was incorporated into M13 phage DNA with two full-length inverted terminal repeats (ITRs), resulting in chimeric virus particles able to achieve stable and specific tumor gene expression [220,221]. When combined with ganciclovir (GCV), this led to effective tumor suppression in laboratory mice and rats, and preclinical tests demonstrated antitumor effects in various cancer models involving cellular apoptosis [221,222,223,224].

Phage-mediated cancer therapy utilizes bacteriophages as delivery vehicles for therapeutic agents, such as cytokines and gene editing tools, with high precision and specificity, reducing off-target effects and minimizing the risk of toxicity to healthy cells. Bacteriophage particles can be engineered to possess functional peptides on their surface to increase their targeting and distribution to cancer cells, thereby improving the efficiency of phage-mediated cancer treatment. The incorporation of an octreotide-targeting ligand improved TNF-selective delivery to pancreatic neuroendocrine tumor cells [225]. By facilitating the endosomal escape of hybrid viral particles containing RGD4C-H5WYG-AAVP, inserting an H5WYG peptide causes cancer cells to undergo apoptosis [226]. Transmorphic phage/AAV particles provide effective delivery of cytokines, including IL-12, IL-15, and TNFα, for selective cytokine therapy against solid tumors. Their compact structure enables successful avoidance of neutralization by immune cells during systemic administration [227]. A recent study combined phage-mediated cancer therapy with clustered, regularly interspaced, short palindrome repeats (CRISPR)/Cas9 genome editing technology and reported the effective co-delivery of CRISPR/Cas9 and a functional *p53* gene to target the mutant *TP53* gene in human lung cancer [228]. This promising approach suggests that phage-mediated cancer therapy is a powerful tool for precision cancer therapy when combined with the CRISPR/Cas9 gene-editing technology.

### 5.3. Combination Therapy of Phages for Targeted Cancer Treatment

Targeted drug delivery is becoming increasingly popular for improving the efficacy of chemotherapy and minimizing its side effects. Phage libraries can identify peptides that bind to cancer cells, allowing direct drug delivery to tumor sites. Phage display-derived peptides are widely used for tumor targeting because of their small size and ease of conjugation with drugs and carriers. Innovative methods of improving chemotherapy are being explored, with promising results. Phages, for instance, have been found to increase the effectiveness of chemotherapy while reducing its side effects [229]. Researchers have also used M13-based phage libraries to identify specific peptides that can penetrate cell membranes and deliver active agents to cancer cells. Two peptide motifs, LTVSPWY and WNLPWYYSVSPT, have been found to bind to breast cancer cells and internalize antisense oligonucleotides [230]. When combined with doxorubicin (DOX), the AGKGTPSLETTP motif from a 12-mer M13-displayed phage library demonstrated strong anticancer efficacy in mice with hepatocarcinoma (HCC) tumors [46]. A pentapeptide phage library identified the ASSHN motif that targets tumor angiogenesis; when conjugated with DOX-loaded liposomes, it showed significant growth inhibition compared with untargeted liposomes [231].

Bacteriophages have also been explored as delivery vehicles for drugs and imaging dyes, with promising results. M13 particles displaying epithelial growth factors can efficiently pack plasmids containing siRNAs against focal adhesion kinases, which effectively target lung carcinoma cells [232]. An anti-prostate-specific membrane antigen (PSMA) antibody was fused with the gp3 protein to create an anti-PSMA-M13-SWNT platform that targets prostate cancer cells and can be used for in vivo fluorescence imaging [87]. Bacteriophages are used in photodynamic therapy to treat cancer. Studies have shown that MS2 bacteriophages decorated with DNA aptamers and M13 bacteriophages targeting breast cancer cells can successfully deliver photosensitizers resulting in cell death [233]. Phage-based nanomedicines using photodynamic therapy can treat cancer by producing singlet oxygen, and a study has shown that genetically modified phages targeting SKBR-3 cancer cells induce cancer cell death upon laser excitation [234]. Moreover, a *Fusobacterium nucleatum* (Fn)-binding M13 phage coated with silver nanoparticles was developed to target Fn in colorectal cancer, which improved mouse survival in an orthotopic colorectal cancer (CRC) model [235].

## 6. Advancement of Phage Use in Humans: Clinical Trials and Patents

Phage displayed antigens have advantages over standard vaccines in activating T cells and stimulating an optimal immune response [75]. Standard vaccinations that employ soluble foreign antigens or inactivated microbes are incapable of activating T cells through the MHC class I pathway, resulting in poor immune responses [236]. Filamentous phages efficiently activate the MHC class I and II pathways, which play a key role in anti-cancer and anti-viral therapies [147,155,212,237]. Phages can also stimulate APCs to secrete costimulatory molecules for T cell activation, making them a promising positive stimulant of the immune system [238]. Phage therapy is being tested globally in clinical trials as a potential treatment for antibiotic-resistant bacterial infections in humans [239]. The American database of clinical trials contains several records related to the use of phages in clinical trials, with a primary focus on the treatment of infectious diseases. However, most studies have focused on the lytic properties of phages to combat antibiotic-resistant bacterial infections. Recently, researchers have investigated the use of phages as a potential treatment for cancer [240,241]. A European phase I/II clinical trial involving patients treated with a phage-based vaccine has been conducted wherein the vaccine linked a selected B-cell receptor to the surface of phage particles. The vaccine was tested in patients with terminal-stage multiple myeloma, and it was well tolerated by patients with minor and transient side effects. The vaccine decreased serum paraprotein and urine-excreted myeloma-specific light chain levels, indicating a clinical response in most patients [242]. Though promising results have been observed in preclinical studies, significant challenges have to be overcome before phage therapy can be widely used in human cancer treatments. Several clinical trials are currently underway to evaluate the safety and efficacy of phage therapy in patients. ABNCoV2, a vaccine utilizing virus-like particles (VLPs) of bacteriophage AP205 decorated with the receptor-binding domain of SARS-CoV-2 and produced in S2 Drosophila cells, was administered to healthy volunteers who were then evaluated for safety in the clinical trial (NCT04839146). Despite promising preclinical results and the safety of phage-based vaccines for humans and animals, the United States Food and Drug Administration (FDA) and The European Medicines Agency (EMA) have not yet approved these vaccines. However, the FDA has approved the use of bacteriophages as antibacterial agents in food products to combat contamination, such as *Listeria monocytogenes* in ready-to-eat meat and poultry (71 FR 47729). Moreover, several patents related to phage therapy for cancer treatment have been granted or are pending approval. These patents include various aspects of phage therapy, such as methods for producing and administering phages, specific phage compositions for targeting cancer cells, and methods for using phages to enhance the antitumor activity of the immune system. Some patents for cancer treatments that utilize phages are listed in Table 3.

## 7. Challenges and Potential of Phage-Based Nanomedicine

Phage-based nanomedicine exhibits potential for use in precision medicine. Table 4 provides an overview of the pros and cons of phages in the biological domains. Manipulating phages to target specific areas could solve the problem of imprecise treatment. However, researchers must address several challenges, such as the underlying mechanisms of the interactions of phages with the immune system and other cells in the body. The efficacy of bacteriophages in entering the body depends on the administration method. When phages enter the bloodstream, they encounter various proteins that signal the immune system for clearance. Oral administration is effective for delivering phages to the gastrointestinal tract, with recovery depending on the dose and age of the individual. However, stomach acidity neutralizers do not significantly improve phage travel through the alimentary tract. Phage replication in the gut outweighs other factors that affect phage recovery efficiency, such as the presence of sensitive bacteria [243]. Moreover, phages can be removed from the body via the liver or kidneys, leading to nonspecific toxicity. Some organs, such as the brain and pancreas, may be difficult for phages to reach, and the fibrotic stroma around tumors may make it difficult for phages to penetrate the tumor [244,245]. This can reduce their efficacy as therapeutic agents.

Phages have a complex multi-component structure that can make them difficult to produce, purify, and standardize compared with small molecule antibiotics. Each phage has a specific host range, and there is a high degree of diversity among phages, making it challenging to find phages that are effective against a wide range of infections. Phages also have the ability to rapidly evolve and adapt to changes in their environment, which can make it challenging to develop long-term therapies. Phage preparations can be contaminated with other bacteria or viruses, which can cause unintended infections in the patient. The long-term effects of phage therapy are not well understood. It may be possible that repeated use of phages could lead to the development of resistance, making future treatments less effective.

Phage-based vaccines can be contaminated with endotoxins, which are lipopolysaccharides that can cause pro-inflammatory reactions, toxic shock, and cell damage even with slight exposure [246]. These endotoxins can originate from the host bacteria, culture media, or be released during the replication of lytic phages [247]. Therefore, it is crucial to remove endotoxins from phage stocks before they can be used in vaccines. Currently, triton X-114 and organic solvents are two effective methods for purifying bacterial cultures of endotoxins [248,249]. Triton X-114 has been shown to efficiently reduce endotoxin concentrations in M13 phage-based vaccinations, which have been well tolerated in clinical studies for patients with minimal flu-like symptoms and skin irritation at the injection site [242].

Regulatory approval of phage therapy is a complex process that requires further clinical trials. Another crucial aspect to consider when exploring the use of phages in cancer immunotherapy is their potential side effects, making it essential to prioritize patient safety. Addressing the considerable problems with production associated with phage long term storage and viability is crucial for the effectiveness of treatment. Combining phages with other drugs, enzymes or particles may help overcome these barriers [46,231,232,233,234,235]. Combining phages with other cancer therapies could enhance their effectiveness; however, further research is required. Despite these challenges, continued research and development in phage-based nanomedicines will lead to exciting advancements in cancer treatments.

## 8. Conclusions

Phages are used in the field of nanomedicine for disease diagnosis and treatment, tissue regeneration, the detection of bacterial and fungal infections, vaccines, and gene therapy. Phages can be used in precision medicine for specific targets. Phage-based vaccines are advantageous over standard vaccines in activating T cells and stimulating an optimal immune response; however, their immunogenicity should be optimized before use. Phage display technology can be used in tumor immunology as a prophylactic and/or therapeutic vaccine or as a small-molecule effector. Since phages can be engineered to be more convenient and accurate carriers for the delivery of therapeutic payloads to cancerous cells, they have emerged as a viable alternative for cancer gene therapy and diagnostics, substituting the vectors based on eukaryotic viruses. It has also been effective in preclinical studies when phage treatment is combined with other drugs, enzymes, or particles. However, researchers must address several challenges, such as understanding the underlying mechanisms of the interactions of phages with the immune system and other cells in the body, and addressing issues such as phage resistance, allergic reactions, and potential unintended side effects. Further research is needed to optimize phage-mediated cancer therapy; however, despite these concerns and challenges, phages exhibit great potential in inducing protective and therapeutic immunity. Overall, continued research and development in phage-based nanomedicines will lead to exciting advancements in cancer treatments, though more studies are needed to fully understand its potential applications and limitations.

## Figures and Tables

**Table 1:** Unique features of the frequently used bacteriophages in phage display research.

Phage Type	Structure	Family	Dimension	Size (kb)	Genome	Coat Protein	Copy Number	Life Cycle
M13	Filamentous	Inoviridae	900 × 7 nm	6.4	ssDNA	pIII, pVIII	5, 2700	Lysogenic
fd	Filamentous	Inoviridae	900 × 7 nm	6.4	ssDNA	pIII, pVIII	5, 2700	Lysogenic
T4	Icosahedral with tail	Myoviridae	120 × 86 nm	168	dsDNA	HOC, SOC	155, 870	Lytic
T7	Icosahedral with tail	Podoviridae	56 × 29 nm	40	dsDNA	gp10B	415	Lytic
λ	Icosahedral with tail	Siphoviridae	60 × 150 nm	48.5	dsDNA	gpD, gpE, gpV	405, 405, 192	Lysogenic/Lytic
MS2	Icosahedral	Leviviridae	26 nm	3.57	ssRNA	Coat protein	178	Lytic
Qβ	Icosahedral	Leviviridae	28 nm	4.2	ssRNA	Coat protein	180	Lytic

**Table 2:** Peptides identified or engineered for cancer therapy using phages.

Peptide Sequence	Target	Biological Applications	Phage/Phage Library	References
DKSEKFARDA	GM-CSF	Colorectal cancer	M13	[113]
CDCRGDCFC	TAA	Breast cancer	T7	[127]
EADPTGHSY	MAGE A1	Antitumor	Fd	[147]
ATWLPPR	VEGF	Anti-angiogenesis	M13	[150]
DTDWVRMRDSAR, VPGWSQAFMALA	EGFR	Lung cancer	Ph.D-12	[151]
HTMYYHHYQHHL	VEGF	Breast carcinoma	Ph.D-12	[152]
KIFGSLAFL	HER2	Tubo tumor	Lambda	[153,154]
GLYDGMEHL, FLWGPRALV	MAGE-A10, MAGE A3	Antitumor	Fd	[155]
LSPPRYP	FGFR	Melanoma	Ph.D-7	[156]
VYMSPF	FGFR1	Anti-angiogenesis	6-mer phage	[157]
ACSLNHTVNC, ACSAKTTSAC	FGFR1	Anti-angiogenesis	Ph.D-C7C	[158]
HSQAAVP	FGF8b	Prostate cancer	Ph.D-7	[159]
QHYNIVNTQSRV	EGFR	Lung carcinoma	M13	[160]
TRTKLPRLHLQS	TAA	Antitumor	M13	[161]
KRTGQYKL	FGFR	Anti-angiogenesis	M13	[162]

**Table 3:** Lists of patents on phage-based cancer treatments (source: USPTO and WIPO/PCT databases).

Patent Number	Inventors	Title	Published Year
WO2006008312A1	Udo Blasi, Christine Hohenadl	Bacteriophage and prophage proteins in cancer gene therapy	2006
US20060058228A1	Kimberly Kelly, David Jones	Colon tumor specific binding peptides	2006
US8137693B2	Valery A. Petrenko	Drug delivery nanocarriers targeted by landscape phage	2012
US8088887B2	Han-Chung, WuChin-Tarng Lin, Tong-Young Lee, Szu-Yao Kuo	Peptide-conjugates that bind to VEGF-stimulated or tumor vasculature and methods of treatment	2012
US8415131B2	Qian Wang, Kai Li, Charlene Mello	M13 bacteriophage as a chemo-addressable nanoparticle for biological and medical applications	2013
US9387257B2	Han-Chung Wu, Yi-Hsuan CHI	Lung cancer specific peptides for targeted drug delivery and molecular imaging	2016
CN105267973A	Wu Hanzhong, Wu Jianxun	Cancer targeting peptides for enhancing anti-cancer drug delivery and therapeutic efficiencies	2016
US9226972B2	Valery A. Petrenko, Deepa Bedi, Olusegun A. Fagbohun, James W. Gillespie	Targeted particles comprising landscape phage fusion proteins and heterologous nucleic acid	2016
US9744223B2	Biswajit Biswas, Carl R. Merril, Hossein A. Ghanbari	Therapeutic cancer vaccine targeted to aspartyl-(asparaginyl)-beta-hydroxylase (HAAH)	2017

**Table 4:** Advantages and disadvantages of phage in the biological domains.

Advantages	Disadvantages
Phage-based nanomedicine can be used for precision medicine	Phages may cause allergic reactions in some individuals
Manipulating phages to target specific areas can solve the problem of imprecise treatment	Limitation in displaying large antigens on phage particles
Phage therapy can potentially reduce the development of antibiotic resistance	Challenging to correctly display a molecule on the phage surface
Strong humoral and cellular immune response without the need for adjuvants	Genome length must be within virion packaging limits for phage DNA vaccines
Can be applied to oral vaccination due to physical stability in the gastrointestinal tract	Efficacy of phages in entering the body depends on the administration method
Phage therapy has the potential to be less expensive than traditional antibiotics	Phage therapy may not be suitable for all patients, such as those with compromised immune systems
Combination therapy with phages is possible	Might be challenging to reach certain organs and tumors to provide effective treatment
Remarkably stable in many challenging environmental conditions	Requires specialized storage and handling
Easy to genetically modify and mass-produce using basic bacteriological media	Endotoxin contamination during phage production
Significant potential for use as vaccine carriers for various illnesses including cancer and infectious diseases	More clinical trials are needed to obtain regulatory approval for phage therapy

## Data Availability

All the data needed to support the conclusions are presented in this paper. Additional data related to this study can be obtained from the authors.

## References

[1] Siegel R.L., Miller K.D., Jemal A. (2019). Cancer statistics, 2019. CA Cancer J. Clin..

[2] Urruticoechea A., Alemany R., Balart J., Villanueva A., Vinals F., Capella G. (2010). Recent advances in cancer therapy: An overview. Curr. Pharm. Des..

[3] Vardy J., Rourke S., Tannock I.F. (2007). Evaluation of cognitive function associated with chemotherapy: A review of published studies and recommendations for future research. J. Clin. Oncol..

[4] Kim Y., Joo K.M., Jin J., Nam D.-H. (2009). Cancer stem cells and their mechanism of chemo-radiation resistance. Int. J. Stem. Cells.

[5] Vardy J., Tannock I. (2007). Cognitive function after chemotherapy in adults with solid tumours. Crit. Rev. Oncol./Hematol..

[6] Amer M.H. (2014). Gene therapy for cancer: Present status and future perspective. Mol. Cell. Ther..

[7] Ailia M.J., Yoo S.Y. (2022). In Vivo Oncolytic Virotherapy in Murine Models of Hepatocellular Carcinoma: A Systematic Review. Vaccines.

[8] Muthukutty P., Woo H.Y., Ragothaman M., Yoo S.Y. (2023). Recent Advances in Cancer Immunotherapy Delivery Modalities. Pharmaceutics.

[9] Agrawal P., Bhalla S., Usmani S.S., Singh S., Chaudhary K., Raghava G.P., Gautam A. (2016). CPPsite 2.0: A repository of experimentally validated cell-penetrating peptides. Nucleic Acids Res..

[10] Arap W., Kolonin M.G., Trepel M., Lahdenranta J., Cardó-Vila M., Giordano R.J., Mintz P.J., Ardelt P.U., Yao V.J., Vidal C.I. (2002). Steps toward mapping the human vasculature by phage display. Nat. Med..

[11] Arap W., Pasqualini R., Ruoslahti E. (1998). Cancer treatment by targeted drug delivery to tumor vasculature in a mouse model. Science.

[12] Bhattarai S.R., Yoo S.Y., Lee S.-W., Dean D. (2012). Engineered phage-based therapeutic materials inhibit Chlamydia trachomatis intracellular infection. Biomaterials.

[13] Chae S.Y., Shrestha K.R., Jeong S.-N., Park G., Yoo S.Y. (2019). Bioinspired RGD-engineered bacteriophage nanofiber cues against oxidative stress. Biomacromolecules.

[14] Lee D.-Y., Lee H., Kim Y., Yoo S.Y., Chung W.-J., Kim G. (2016). Phage as versatile nanoink for printing 3-D cell-laden scaffolds. Acta Biomater..

[15] Lee H.-S., Kang J.-I., Chung W.-J., Lee D.H., Lee B.Y., Lee S.-W., Yoo S.Y. (2018). Engineered phage matrix stiffness-modulating osteogenic differentiation. ACS Appl. Mater. Interfaces.

[16] Lee J.H., Kim S.W., Ji S.T., Kim Y.J., Jang W.B., Oh J.-W., Kim J., Yoo S.Y., Beak S.H., Kwon S.-M. (2017). Engineered M13 nanofiber accelerates ischemic neovascularization by enhancing endothelial progenitor cells. Tissue Eng. Regen. Med..

[17] Moon J.-S., Kim W.-G., Kim C., Park G.-T., Heo J., Yoo S.Y., Oh J.-W. (2015). M13 bacteriophage-based self-assembly structures and their functional capabilities. Mini-Rev. Org. Chem..

[18] Shrestha K.R., Lee D.H., Chung W., Lee S.-W., Lee B.Y., Yoo S.Y. (2022). Biomimetic virus-based soft niche for ischemic diseases. Biomaterials.

[19] Sugimoto R., Lee J.H., Lee J.-H., Jin H.-E., Yoo S.Y., Lee S.-W. (2019). Bacteriophage nanofiber fabrication using near field electrospinning. RSC Adv..

[20] Yoo S.Y., Chung W.-J., Kim T.H., Le M., Lee S.-W. (2011). Facile patterning of genetically engineered M13 bacteriophage for directional growth of human fibroblast cells. Soft Matter.

[21] Yoo S.Y., Jin H.E., Choi D.S., Kobayashi M., Farouz Y., Wang S., Lee S.W. (2016). M13 bacteriophage and adeno-associated virus hybrid for novel tissue engineering material with gene delivery functions. Adv. Healthc. Mater..

[22] Yoo S.Y., Kobayashi M., Lee P.P., Lee S.-W. (2011). Early osteogenic differentiation of mouse preosteoblasts induced by collagen-derived DGEA-peptide on nanofibrous phage tissue matrices. Biomacromolecules.

[23] Yoo S.Y., Merzlyak A., Lee S.-W. (2011). Facile growth factor immobilization platform based on engineered phage matrices. Soft Matter.

[24] Yoo S.Y., Merzlyak A., Lee S.-W. (2014). Synthetic phage for tissue regeneration. Mediat. Inflamm..

[25] Yoo S.Y., Shrestha K.R., Jeong S.-N., Kang J.-I., Lee S.-W. (2017). Engineered phage nanofibers induce angiogenesis. Nanoscale.

[26] Moon J.-S., Kim W.-G., Shin D.-M., Lee S.-Y., Kim C., Lee Y., Han J., Kim K., Yoo S.Y., Oh J.-W. (2017). Bioinspired M-13 bacteriophage-based photonic nose for differential cell recognition. Chem. Sci..

[27] Au G.G., Lincz L.F., Enno A., Shafren D.R. (2007). Oncolytic Coxsackievirus A21 as a novel therapy for multiple myeloma. Br. J. Haematol..

[28] Bachrach G., Leizerovici-Zigmond M., Zlotkin A., Naor R., Steinberg D. (2003). Bacteriophage isolation from human saliva. Lett. Appl. Microbiol..

[29] Bais S., Bartee E., Rahman M.M., McFadden G., Cogle C.R. (2012). Oncolytic virotherapy for hematological malignancies. Adv. Virol..

[30] Bearden C.M., Agarwal A., Book B.K., Vieira C.A., Sidner R.A., Ochs H.D., Young M., Pescovitz M.D. (2005). Rituximab inhibits the in vivo primary and secondary antibody response to a neoantigen, bacteriophage phiX174. Am. J. Transplant..

[31] Burrows F.J., Thorpe P.E. (1994). Vascular targeting—A new approach to the therapy of solid tumors. Pharmacol. Ther..

[32] Bazan J., Całkosiński I., Gamian A. (2012). Phage display—A powerful technique for immunotherapy: 1. Introduction and potential of therapeutic applications. Hum. Vaccines Immunother..

[33] Hajitou A. (2010). Targeted systemic gene therapy and molecular imaging of cancer: Contribution of the vascular-targeted AAVP vector. Adv. Genet..

[34] Clark J.R., March J.B. (2004). Bacterial viruses as human vaccines?. Expert. Rev. Vaccines.

[35] Twort F.W. (1936). Further investigations on the nature of ultra-microscopic viruses and their cultivation. Epidemiol. Infect..

[36] Taylor M.W. (2014). Viruses and Man: A History of Interactions.

[37] Schless R.A. (1932). Staphylococcus aureus meningitis: Treatment with specific bacteriophage. Am. J. Dis. Child..

[38] Summers W.C.S. (1999). Felix D’herelle and the Origins of Molecular Biology.

[39] Rice T.B. (1930). Use of bacteriophage filtrates in treatment of suppurative conditions: Report of 300 cases. Am. J. Med. Sci..

[40] De la Cruz V., Lal A., McCutchan T.F. (1988). Immunogenicity and epitope mapping of foreign sequences via genetically engineered filamentous phage. J. Biol. Chem..

[41] Bartolacci C., Andreani C., Curcio C., Occhipinti S., Massaccesi L., Giovarelli M., Galeazzi R., Iezzi M., Tilio M., Gambini V. (2018). Phage-Based Anti-HER2 Vaccination Can Circumvent Immune Tolerance against Breast CancerPhages against HER2+ Breast Cancer. Cancer Immunol. Res..

[42] Asadi-Ghalehni M., Ghaemmaghami M., Klimka A., Javanmardi M., Navari M., Rasaee M.J. (2015). Cancer immunotherapy by a recombinant phage vaccine displaying EGFR mimotope: An in vivo study. Immunopharmacol. Immunotoxicol..

[43] Adhya S., Merril C.R., Biswas B. (2014). Therapeutic and prophylactic applications of bacteriophage components in modern medicine. Cold Spring Harb. Perspect. Med..

[44] Liu A., Abbineni G., Mao C. (2009). Nanocomposite films assembled from genetically engineered filamentous viruses and gold nanoparticles: Nanoarchitecture-and humidity-tunable surface plasmon resonance spectra. Adv. Mater..

[45] Larocca D., Witte A., Johnson W., Pierce G.F., Baird A. (1998). Targeting bacteriophage to mammalian cell surface receptors for gene delivery. Hum. Gene Ther..

[46] Du B., Han H., Wang Z., Kuang L., Wang L., Yu L., Wu M., Zhou Z., Qian M. (2010). Targeted Drug Delivery to Hepatocarcinoma In vivo by Phage-Displayed Specific Binding PeptideTargeted Drug Delivery to Hepatocarcinoma by Peptide. Mol. Cancer Res..

[47] Hatfull G.F., Hendrix R.W. (2011). Bacteriophages and their genomes. Curr. Opin. Virol..

[48] Kostyuchenko V.A., Leiman P.G., Chipman P.R., Kanamaru S., Van Raaij M.J., Arisaka F., Mesyanzhinov V.V., Rossmann M.G. (2003). Three-dimensional structure of bacteriophage T4 baseplate. Nat. Struct. Mol. Biol..

[49] Straus S.K., Bo H.E. (2018). Filamentous bacteriophage proteins and assembly. Virus Protein Nucl. Complexes.

[50] Dams D., Brøndsted L., Drulis-Kawa Z., Briers Y. (2019). Engineering of receptor-binding proteins in bacteriophages and phage tail-like bacteriocins. Biochem. Soc. Trans..

[51] Maghsoodi A., Chatterjee A., Andricioaei I., Perkins N.C. (2019). How the phage T4 injection machinery works including energetics, forces, and dynamic pathway. Proc. Natl. Acad. Sci. USA.

[52] Hay I.D., Lithgow T. (2019). Filamentous phages: Masters of a microbial sharing economy. EMBO Rep..

[53] Mäntynen S., Laanto E., Oksanen H.M., Poranen M.M., Díaz-Muñoz S.L. (2021). Black box of phage–bacterium interactions: Exploring alternative phage infection strategies. Open Biol..

[54] Olszak T., Latka A., Roszniowski B., Valvano M.A., Drulis-Kawa Z. (2017). Phage life cycles behind bacterial biodiversity. Curr. Med. Chem..

[55] Żaczek M., Weber-Dąbrowska B., Międzybrodzki R., Górski A. (2020). Phage prevalence in the human urinary tract—Current knowledge and therapeutic implications. Microorganisms.

[56] Manrique P., Dills M., Young M.J. (2017). The human gut phage community and its implications for health and disease. Viruses.

[57] Huh H., Wong S., Jean J.S., Slavcev R. (2019). Bacteriophage interactions with mammalian tissue: Therapeutic applications. Adv. Drug. Deliv. Rev..

[58] Nguyen S., Baker K., Padman B.S., Patwa R., Dunstan R.A., Weston T.A., Schlosser K., Bailey B., Lithgow T., Lazarou M. (2017). Bacteriophage transcytosis provides a mechanism to cross epithelial cell layers. MBio.

[59] Karimi M., Mirshekari H., Basri S.M.M., Bahrami S., Moghoofei M., Hamblin M.R. (2016). Bacteriophages and phage-inspired nanocarriers for targeted delivery of therapeutic cargos. Adv. Drug Deliv. Rev..

[60] Parent K.N., Schrad J.R., Cingolani G. (2018). Breaking symmetry in viral icosahedral capsids as seen through the lenses of X-ray crystallography and cryo-electron microscopy. Viruses.

[61] Louten J. (2016). Virus Structure and Classification. Essent. Hum. Virol..

[62] Han J.-H., Wang M.S., Das J., Sudheendra L., Vonasek E., Nitin N., Kennedy I.M. (2014). Capture and detection of T7 bacteriophages on a nanostructured interface. ACS Appl. Mater. Interfaces.

[63] Passaretti P., Sun Y., Dafforn T.R., Oppenheimer P.G. (2020). Determination and characterisation of the surface charge properties of the bacteriophage M13 to assist bio-nanoengineering. RSC Adv..

[64] Golec P., Dąbrowski K., Hejnowicz M.S., Gozdek A., Łoś J.M., Węgrzyn G., Łobocka M.B., Łoś M. (2011). A reliable method for storage of tailed phages. J. Microbiol. Methods.

[65] Jepson C.D., March J.B. (2004). Bacteriophage lambda is a highly stable DNA vaccine delivery vehicle. Vaccine.

[66] Brigati J.R., Petrenko V.A. (2005). Thermostability of landscape phage probes. Anal. Bioanal. Chem..

[67] Duyvejonck H., Merabishvili M., Vaneechoutte M., De Soir S., Wright R., Friman V.-P., Verbeken G., De Vos D., Pirnay J.-P., Van Mechelen E. (2021). Evaluation of the stability of bacteriophages in different solutions suitable for the production of magistral preparations in Belgium. Viruses.

[68] Górski A., Międzybrodzki R., Borysowski J., Dąbrowska K., Wierzbicki P., Ohams M., Korczak-Kowalska G., Olszowska-Zaremba N., Łusiak-Szelachowska M., Kłak M. (2012). Phage as a modulator of immune responses: Practical implications for phage therapy. Adv. Virus Res..

[69] Oakes R.S., Froimchuk E., Jewell C.M. (2019). Engineering biomaterials to direct innate immunity. Adv. Ther..

[70] Chang T.Z., Diambou I., Kim J.R., Wang B., Champion J.A. (2017). Host-and pathogen-derived adjuvant coatings on protein nanoparticle vaccines. Bioeng. Transl. Med..

[71] Iwagami Y., Casulli S., Nagaoka K., Kim M., Carlson R.I., Ogawa K., Lebowitz M.S., Fuller S., Biswas B., Stewart S. (2017). Lambda phage-based vaccine induces antitumor immunity in hepatocellular carcinoma. Heliyon.

[72] Dong X., Pan P., Ye J.-J., Zhang Q.-L., Zhang X.-Z. (2022). Hybrid M13 bacteriophage-based vaccine platform for personalized cancer immunotherapy. Biomaterials.

[73] Samoylov A., Cochran A., Schemera B., Kutzler M., Donovan C., Petrenko V., Bartol F., Samoylova T. (2015). Humoral immune responses against gonadotropin releasing hormone elicited by immunization with phage-peptide constructs obtained via phage display. J. Biotechnol..

[74] Palma M. (2023). Aspects of Phage-Based Vaccines for Protein and Epitope Immunization. Vaccines.

[75] González-Mora A., Hernández-Pérez J., Iqbal H.M., Rito-Palomares M., Benavides J. (2020). Bacteriophage-based vaccines: A potent approach for antigen delivery. Vaccines.

[76] Tao P., Zhu J., Mahalingam M., Batra H., Rao V.B. (2019). Bacteriophage T4 nanoparticles for vaccine delivery against infectious diseases. Adv. Drug Deliv. Rev..

[77] Hamzeh-Mivehroud M., Mahmoudpour A., Rezazadeh H., Dastmalchi S. (2008). Non-specific translocation of peptide-displaying bacteriophage particles across the gastrointestinal barrier. Eur. J. Pharm. Biopharm..

[78] Duerr D.M., White S.J., Schluesener H.J. (2004). Identification of peptide sequences that induce the transport of phage across the gastrointestinal mucosal barrier. J. Virol. Methods.

[79] Aghebati-Maleki L., Bakhshinejad B., Baradaran B., Motallebnezhad M., Aghebati-Maleki A., Nickho H., Yousefi M., Majidi J. (2016). Phage display as a promising approach for vaccine development. J. Biomed. Sci..

[80] Febvre H.P., Rao S., Gindin M., Goodwin N.D., Finer E., Vivanco J.S., Lu S., Manter D.K., Wallace T.C., Weir T.L. (2019). PHAGE study: Effects of supplemental bacteriophage intake on inflammation and gut microbiota in healthy adults. Nutrients.

[81] Laforêt F., Antoine C., Lebrun S., Gonza I., Goya-Jorge E., Douny C., Duprez J.-N., Scippo M.-L., Taminiau B., Daube G. (2023). Impact Assessment of vB_KpnP_K1-ULIP33 Bacteriophage on the Human Gut Microbiota Using a Dynamic In Vitro Model. Viruses.

[82] Delmastro P., Meola A., Monaci P., Cortese R., Galfre G. (1997). Immunogenicity of filamentous phage displaying peptide mimotopes after oral administration. Vaccine.

[83] Ren Z., Tian C., Zhu Q., Zhao M., Xin A., Nie W., Ling S., Zhu M., Wu J., Lan H. (2008). Orally delivered foot-and-mouth disease virus capsid protomer vaccine displayed on T4 bacteriophage surface: 100% protection from potency challenge in mice. Vaccine.

[84] Hess K.L., Jewell C.M. (2020). Phage display as a tool for vaccine and immunotherapy development. Bioeng. Transl. Med..

[85] Gray B.P., Brown K.C. (2014). Combinatorial peptide libraries: Mining for cell-binding peptides. Chem. Rev..

[86] Rakonjac J., Bennett N.J., Spagnuolo J., Gagic D., Russel M. (2011). Filamentous bacteriophage: Biology, phage display and nanotechnology applications. Curr. Issues Mol. Biol..

[87] Yi H., Ghosh D., Ham M.-H., Qi J., Barone P.W., Strano M.S., Belcher A.M. (2012). M13 phage-functionalized single-walled carbon nanotubes as nanoprobes for second near-infrared window fluorescence imaging of targeted tumors. Nano Lett..

[88] Ahmad G., Dickerson M.B., Church B.C., Cai Y., Jones S.E., Naik R.R., King J.S., Summers C.J., Kröger N., Sandhage K.H. (2006). Rapid, room-temperature formation of crystalline calcium molybdate phosphor microparticles via peptide-induced precipitation. Adv. Mater..

[89] Barry M.A., Dower W.J., Johnston S.A. (1996). Toward cell–targeting gene therapy vectors: Selection of cell–binding peptides from random peptide–presenting phage libraries. Nat. Med..

[90] Li J., Feng L., Fan L., Zha Y., Guo L., Zhang Q., Chen J., Pang Z., Wang Y., Jiang X. (2011). Targeting the brain with PEG–PLGA nanoparticles modified with phage-displayed peptides. Biomaterials.

[91] Abbineni G., Modali S., Safiejko-Mroczka B., Petrenko V.A., Mao C. (2010). Evolutionary selection of new breast cancer cell-targeting peptides and phages with the cell-targeting peptides fully displayed on the major coat and their effects on actin dynamics during cell internalization. Mol. Pharm..

[92] Straus S., Scott W., Symmons M., Marvin D. (2008). On the structures of filamentous bacteriophage Ff (fd, f1, M13). Eur. Biophys. J..

[93] Yang S.H., Chung W.J., McFarland S., Lee S.W. (2013). Assembly of bacteriophage into functional materials. Chem. Rec..

[94] Rajotte D., Arap W., Hagedorn M., Koivunen E., Pasqualini R., Ruoslahti E. (1998). Molecular heterogeneity of the vascular endothelium revealed by in vivo phage display. J. Clin. Investig..

[95] Kobayashi N., Oyama H., Nakano M., Kanda T., Banzono E., Kato Y., Karibe T., Nishio T., Goto J. (2009). “Cleavable” hapten–biotin conjugates: Preparation and use for the generation of anti-steroid single-domain antibody fragments. Anal. Biochem..

[96] Even-Desrumeaux K., Nevoltris D., Lavaut M.N., Alim K., Borg J.-P., Audebert S., Kerfelec B., Baty D., Chames P. (2014). Masked selection: A straightforward and flexible approach for the selection of binders against specific epitopes and differentially expressed proteins by phage display. Mol. Cell. Proteom..

[97] Zheng H.-M., Jiang Y., Wang J.-R., Gong X.-L., Guo B.-Y. (2011). Mimic peptides bonding specifically with the first and second extracellular loops of the CC chemokine receptor 5 derived from a phage display peptide library are potent inhibitors of experimental autoimmune encephalomyelitis. Inflamm. Res..

[98] Blanchetot C., Verzijl D., Mujić-Delić A., Bosch L., Rem L., Leurs R., Verrips C.T., Saunders M., de Haard H., Smit M.J. (2013). Neutralizing nanobodies targeting diverse chemokines effectively inhibit chemokine function. J. Biol. Chem..

[99] Yanofsky S.D., Baldwin D.N., Butler J.H., Holden F.R., Jacobs J.W., Balasubramanian P., Chinn J.P., Cwirla S.E., Peters-Bhatt E., Whitehorn E.A. (1996). High affinity type I interleukin 1 receptor antagonists discovered by screening recombinant peptide libraries. Proc. Natl. Acad. Sci. USA.

[100] Merzlyak A., Indrakanti S., Lee S.-W. (2009). Genetically engineered nanofiber-like viruses for tissue regenerating materials. Nano Lett..

[101] Merzlyak A., Lee S.-W. (2009). Engineering phage materials with desired peptide display: Rational design sustained through natural selection. Bioconjugate Chem..

[102] Lee S.-W., Mao C., Flynn C.E., Belcher A.M. (2002). Ordering of quantum dots using genetically engineered viruses. Science.

[103] Lee S.-W., Wood B.M., Belcher A.M. (2003). Chiral smectic C structures of virus-based films. Langmuir.

[104] Merzlyak A., Lee S.-W. (2006). Phage as templates for hybrid materials and mediators for nanomaterial synthesis. Curr. Opin. Chem. Biol..

[105] Naik R.R., Stringer S.J., Agarwal G., Jones S.E., Stone M.O. (2002). Biomimetic synthesis and patterning of silver nanoparticles. Nat. Mater..

[106] Bermudez H., Hathorne A.P. (2008). Incorporating stimulus-responsive character into filamentous virus assemblies. Faraday Discuss..

[107] Rohovie M.J., Nagasawa M., Swartz J.R. (2017). Virus-like particles: Next-generation nanoparticles for targeted therapeutic delivery. Bioeng. Transl. Med..

[108] Nagano K., Tsutsumi Y. (2016). Development of novel drug delivery systems using phage display technology for clinical application of protein drugs. Proc. Jpn. Acad. Ser. B.

[109] Bakhshinejad B., Sadeghizadeh M. (2014). Bacteriophages and development of nanomaterials for neural regeneration. Neural Regen. Res..

[110] Sorokulova I., Olsen E., Vodyanoy V. (2014). Bacteriophage biosensors for antibiotic-resistant bacteria. Expert. Rev. Med. Devices.

[111] Schmelcher M., Loessner M.J. (2014). Application of bacteriophages for detection of foodborne pathogens. Bacteriophage.

[112] Li S., Li Y., Chen H., Horikawa S., Shen W., Simonian A., Chin B.A. (2010). Direct detection of Salmonella typhimurium on fresh produce using phage-based magnetoelastic biosensors. Biosens. Bioelectron..

[113] Wang H.Y., Chang Y.-C., Hu C.-W., Kao C.-Y., Yu Y.-A., Lim S.-K., Mou K.Y. (2021). Development of a Novel Cytokine Vehicle Using Filamentous Phage Display for Colorectal Cancer Treatment. ACS Synth. Biol..

[114] Jin H.-E., Farr R., Lee S.-W. (2014). Collagen mimetic peptide engineered M13 bacteriophage for collagen targeting and imaging in cancer. Biomaterials.

[115] Fokine A., Islam M.Z., Zhang Z., Bowman V.D., Rao V.B., Rossmann M.G. (2011). Structure of the three N-terminal immunoglobulin domains of the highly immunogenic outer capsid protein from a T4-like bacteriophage. J. Virol..

[116] Qin L., Fokine A., O’Donnell E., Rao V.B., Rossmann M.G. (2010). Structure of the small outer capsid protein, Soc: A clamp for stabilizing capsids of T4-like phages. J. Mol. Biol..

[117] Ren Z.-J., Black L.W. (1998). Phage T4 SOC and HOC display of biologically active, full-length proteins on the viral capsid. Gene.

[118] Tao P., Mahalingam M., Kirtley M.L., van Lier C.J., Sha J., Yeager L.A., Chopra A.K., Rao V.B. (2013). Mutated and bacteriophage T4 nanoparticle arrayed F1-V immunogens from Yersinia pestis as next generation plague vaccines. PLoS Pathog..

[119] Li Q., Shivachandra S.B., Zhang Z., Rao V.B. (2007). Assembly of the small outer capsid protein, Soc, on bacteriophage T4: A novel system for high density display of multiple large anthrax toxins and foreign proteins on phage capsid. J. Mol. Biol..

[120] Gamkrelidze M., Dąbrowska K. (2014). T4 bacteriophage as a phage display platform. Arch. Microbiol..

[121] Dąbrowska K., Miernikiewicz P., Piotrowicz A., Hodyra K., Owczarek B., Lecion D., Kaźmierczak Z., Letarov A., Górski A. (2014). Immunogenicity studies of proteins forming the T4 phage head surface. J. Virol..

[122] Sanmukh S.G., Santos N.J., Barquilha C.N., Dos Santos S.A.A., Duran B.O.S., Delella F.K., Moroz A., Justulin L.A., Carvalho H.F., Felisbino S.L. (2021). Exposure to Bacteriophages T4 and M13 Increases Integrin Gene Expression and Impairs Migration of Human PC-3 Prostate Cancer Cells. Antibiotics.

[123] Danner S., Belasco J.G. (2001). T7 phage display: A novel genetic selection system for cloning RNA-binding proteins from cDNA libraries. Proc. Natl. Acad. Sci. USA.

[124] Xu H., Bao X., Wang Y., Xu Y., Deng B., Lu Y., Hou J. (2018). Engineering T7 bacteriophage as a potential DNA vaccine targeting delivery vector. Virol. J..

[125] Eguchi A., Akuta T., Okuyama H., Senda T., Yokoi H., Inokuchi H., Fujita S., Hayakawa T., Takeda K., Hasegawa M. (2001). Protein transduction domain of HIV-1 Tat protein promotes efficient delivery of DNA into mammalian cells. J. Biol. Chem..

[126] Kim A., Shin T.-H., Shin S.-M., Pham C.D., Choi D.-K., Kwon M.-H., Kim Y.-S. (2012). Cellular internalization mechanism and intracellular trafficking of filamentous M13 phages displaying a cell-penetrating transbody and TAT peptide. PLoS ONE.

[127] Dasa S.S.K., Jin Q., Chen C.-T., Chen L. (2012). Target-specific copper hybrid T7 phage particles. Langmuir.

[128] Cicchini C., Ansuini H., Amicone L., Alonzi T., Nicosia A., Cortese R., Tripodi M., Luzzago A. (2002). Searching for DNA–protein interactions by lambda phage display. J. Mol. Biol..

[129] Santi E., Capone S., Mennuni C., Lahm A., Tramontano A., Luzzago A., Nicosia A. (2000). Bacteriophage lambda display of complex cDNA libraries: A new approach to functional genomics. J. Mol. Biol..

[130] Santini C., Brennan D., Mennuni C., Hoess R.H., Nicosia A., Cortese R., Luzzago A. (1998). Eficient display of an HCV cDNA expression library as C-terminal fusion to the capsid protein D of bacteriophage lambda. J. Mol. Biol..

[131] Mikawa Y.G., Maruyama I.N., Brenner S. (1996). Surface display of proteins on bacteriophage λ heads. J. Mol. Biol..

[132] Sternberg N., HoEss R.H. (1995). Display of peptides and proteins on the surface of bacteriophage lambda. Proc. Natl. Acad. Sci. USA.

[133] Kuwabara I., Maruyama H., Mikawa Y.G., Zuberi R.I., Liu F.-T., Maruyama I.N. (1997). Efficient epitope mapping by bacteriophage λ surface display. Nat. Biotechnol..

[134] Maruyama I.N., Maruyama H.I., Brenner S. (1994). Lambda foo: A lambda phage vector for the expression of foreign proteins. Proc. Natl. Acad. Sci. USA.

[135] Lankes H., Zanghi C., Santos K., Capella C., Duke C., Dewhurst S. (2007). In vivo gene delivery and expression by bacteriophage lambda vectors. J. Appl. Microbiol..

[136] Razazan A., Nicastro J., Slavcev R., Barati N., Arab A., Mosaffa F., Jaafari M.R., Behravan J. (2019). Lambda bacteriophage nanoparticles displaying GP2, a HER2/neu derived peptide, induce prophylactic and therapeutic activities against TUBO tumor model in mice. Sci. Rep..

[137] Catala A., Dzieciatkowska M., Wang G., Gutierrez-Hartmann A., Simberg D., Hansen K.C., D’Alessandro A., Catalano C.E. (2021). Targeted intracellular delivery of trastuzumab using designer phage lambda nanoparticles alters cellular programs in human breast cancer cells. ACS Nano.

[138] Bookstaver M.L., Tsai S.J., Bromberg J.S., Jewell C.M. (2018). Improving vaccine and immunotherapy design using biomaterials. Trends Immunol..

[139] Sanmukh S.G., Dos Santos N.J., Barquilha C.N., De Carvalho M., Dos Reis P.P., Delella F.K., Carvalho H.F., Latek D., Fehér T., Felisbino S.L. (2023). Bacterial RNA virus MS2 exposure increases the expression of cancer progression genes in the LNCaP prostate cancer cell line. Oncol. Lett..

[140] Reed C.A., Langlais C., Wang I.-N., Young R. (2013). A2 expression and assembly regulates lysis in Qβ infections. Microbiology.

[141] Nchinda G.W., Al-Atoom N., Coats M.T., Cameron J.M., Waffo A.B. (2021). Uniqueness of RNA coliphage Qβ Display system in directed evolutionary biotechnology. Viruses.

[142] Rumnieks J., Tars K. (2011). Crystal structure of the read-through domain from bacteriophage Qβ A1 protein. Protein Sci..

[143] Yin Z., Comellas-Aragones M., Chowdhury S., Bentley P., Kaczanowska K., BenMohamed L., Gildersleeve J.C., Finn M., Huang X. (2013). Boosting immunity to small tumor-associated carbohydrates with bacteriophage Qβ capsids. ACS Chem. Biol..

[144] Ren S., Zuo S., Zhao M., Wang X., Wang X., Chen Y., Wu Z., Ren Z. (2011). Inhibition of tumor angiogenesis in lung cancer by T4 phage surface displaying mVEGFR2 vaccine. Vaccine.

[145] Barati N., Razazan A., Nicastro J., Slavcev R., Arab A., Mosaffa F., Nikpoor A.R., Badiee A., Jaafari M.R., Behravan J. (2018). Immunogenicity and antitumor activity of the superlytic λF7 phage nanoparticles displaying a HER2/neu-derived peptide AE37 in a tumor model of BALB/c mice. Cancer Lett..

[146] Liu D., Tang L., Zhou C., Tan L. (2006). Immunotherapy of EGFR-positive tumor based on recombinant EGFR phage vaccine. Chin.-Ger. J. Clin. Oncol..

[147] Fang J., Wang G., Yang Q., Song J., Wang Y., Wang L. (2005). The potential of phage display virions expressing malignant tumor specific antigen MAGE-A1 epitope in murine model. Vaccine.

[148] Yin Z., Wu X., Kaczanowska K., Sungsuwan S., Comellas Aragones M., Pett C., Yu J., Baniel C., Westerlind U., Finn M. (2018). Antitumor humoral and T cell responses by mucin-1 conjugates of bacteriophage Qβ in wild-type mice. ACS Chem. Biol..

[149] Ren S.-x., Ren Z.-j., Zhao M.-y., Wang X.-b., Zuo S.-g., Yu F. (2009). Antitumor activity of endogenous mFlt4 displayed on a T4 phage nanoparticle surface. Acta Pharmacol. Sin..

[150] Binetruy-Tournaire R., Demangel C., Malavaud B., Vassy R., Rouyre S., Kraemer M., Plouet J., Derbin C., Perret G., Mazie J.C. (2000). Identification of a peptide blocking vascular endothelial growth factor (VEGF)-mediated angiogenesis. EMBO J..

[151] Wang A., Cui M., Qu H., Di J., Wang Z., Xing J., Wu F., Wu W., Wang X., Shen L. (2016). Induction of anti-EGFR immune response with mimotopes identified from a phage display peptide library by panitumumab. Oncotarget.

[152] Hetian L., Ping A., Shumei S., Xiaoying L., Luowen H., Jian W., Lin M., Meisheng L., Junshan Y., Chengchao S. (2002). A novel peptide isolated from a phage display library inhibits tumor growth and metastasis by blocking the binding of vascular endothelial growth factor to its kinase domain receptor. J. Biol. Chem..

[153] Arab A., Nicastro J., Slavcev R., Razazan A., Barati N., Nikpoor A.R., Brojeni A.A.M., Mosaffa F., Badiee A., Jaafari M.R. (2018). Lambda phage nanoparticles displaying HER2-derived E75 peptide induce effective E75-CD8+ T response. Immunol. Res..

[154] Clifton G.T., Peoples G.E., Mittendorf E.A. (2016). The development and use of the E75 (HER2 369–377) peptide vaccine. Future Oncol..

[155] Sartorius R., Pisu P., D’Apice L., Pizzella L., Romano C., Cortese G., Giorgini A., Santoni A., Velotti F., De Berardinis P. (2008). The use of filamentous bacteriophage fd to deliver MAGE-A10 or MAGE-A3 HLA-A2-restricted peptides and to induce strong antitumor CTL responses. J. Immunol..

[156] Wu X., Huang H., Wang C., Lin S., Huang Y., Wang Y., Liang G., Yan Q., Xiao J., Wu J. (2013). Identification of a novel peptide that blocks basic fibroblast growth factor-mediated cell proliferation. Oncotarget.

[157] Fan H., Duan Y., Zhou H., Li W., Li F., Guo L., Roeske R.W. (2002). Selection of peptide ligands binding to fibroblast growth factor receptor 1. IUBMB Life.

[158] Lipok M., Szlachcic A., Kindela K., Czyrek A., Otlewski J. (2019). Identification of a peptide antagonist of the FGF 1–FGFR 1 signaling axis by phage display selection. FEBS Open Bio..

[159] Wang W., Chen X., Li T., Li Y., Wang R., He D., Luo W., Li X., Wu X. (2013). Screening a phage display library for a novel FGF8b-binding peptide with anti-tumor effect on prostate cancer. Exp. Cell. Res..

[160] Asadi-Ghalehni M., Rasaee M.J., Asl N.N., Khosravani M., Rajabibazl M., Modjtahedi H., Sadroddiny E. (2018). Construction of a recombinant phage-vaccine capable of reducing the growth rate of an established LL2 tumor model. Iran. J. Allergy Asthma Immunol..

[161] Eriksson F., Culp W.D., Massey R., Egevad L., Garland D., Persson M.A., Pisa P. (2007). Tumor specific phage particles promote tumor regression in a mouse melanoma model. Cancer Immunol. Immunother..

[162] Yayon A., Aviezer D., Safran M., Gross J.L., Heldman Y., Cabilly S., Givol D., Katchalski-Katzir E. (1993). Isolation of peptides that inhibit binding of basic fibroblast growth factor to its receptor from a random phage-epitope library. Proc. Natl. Acad. Sci. USA.

[163] Wu C.-H., Liu I.-J., Lu R.-M., Wu H.-C. (2016). Advancement and applications of peptide phage display technology in biomedical science. J. Biomed. Sci..

[164] An P., Lei H., Zhang J., Song S., He L., Jin G., Liu X., Wu J., Meng L., Liu M. (2004). Suppression of tumor growth and metastasis by a VEGFR-1 antagonizing peptide identified from a phage display library. Int. J. Cancer.

[165] Pardoll D. (2015). Cancer and the immune system: Basic concepts and targets for intervention. Semin. Oncol..

[166] Zuo S., Dai G., Wang L., Wen Y., Huang Z., Yang W., Ma W., Ren X. (2019). Suppression of angiogenesis and tumor growth by recombinant T4 phages displaying extracellular domain of vascular endothelial growth factor receptor 2. Arch. Virol..

[167] Hurwitz H. (2004). Integrating the anti–VEGF-A humanized monoclonal antibody bevacizumab with chemotherapy in advanced colorectal cancer. Clin. Color. Cancer.

[168] Zhang J., Li H., Wang X., Qi H., Miao X., Zhang T., Chen G., Wang M. (2012). Phage-derived fully human antibody scFv fragment directed against human vascular endothelial growth factor receptor 2 blocked its interaction with VEGF. Biotechnol. Prog..

[169] Lamdan H., Gavilondo J.V., Munoz Y., Pupo A., Huerta V., Musacchio A., Pérez L., Ayala M., Rojas G., Balint R.F. (2013). Affinity maturation and fine functional mapping of an antibody fragment against a novel neutralizing epitope on human vascular endothelial growth factor. Mol. BioSyst..

[170] Kordi S., Rahmati-Yamchi M., Vostakolaei M.A., Etemadie A., Barzegari A., Abdolalizadeh J. (2019). Isolation of a novel anti-kdr3 single-chain variable fragment antibody from a phage display library. Iran. J. Allergy Asthma Immunol..

[171] Giordano R.J., Cardó-Vila M., Salameh A., Anobom C.D., Zeitlin B.D., Hawke D.H., Valente A.P., Almeida F.C., Nör J.E., Sidman R.L. (2010). From combinatorial peptide selection to drug prototype (I): Targeting the vascular endothelial growth factor receptor pathway. Proc. Natl. Acad. Sci. USA.

[172] Lu L., Chen H., Hao D., Zhang X., Wang F. (2019). The functions and applications of A7R in anti-angiogenic therapy, imaging and drug delivery systems. Asian J. Pharm. Sci..

[173] Giordano R.J., Cardó-Vila M., Lahdenranta J., Pasqualini R., Arap W. (2001). Biopanning and Rapid Analysis of Selective Interactive Ligands.

[174] Roskoski R. (2014). The ErbB/HER family of protein-tyrosine kinases and cancer. Pharmacol. Res..

[175] Engelman J.A., Cantley L.C. (2008). A sweet new role for EGFR in cancer. Cancer Cell.

[176] Rajaram P., Chandra P., Ticku S., Pallavi B., Rudresh K., Mansabdar P. (2017). Epidermal growth factor receptor: Role in human cancer. Indian J. Dent. Res..

[177] Yavari B., Mahjub R., Saidijam M., Raigani M., Soleimani M. (2018). The potential use of peptides in cancer treatment. Curr. Protein Pept. Sci..

[178] Roovers R.C., Laeremans T., Huang L., De Taeye S., Verkleij A.J., Revets H., de Haard H.J., van Bergen en Henegouwen P.M.P. (2007). Efficient inhibition of EGFR signalling and of tumour growth by antagonistic anti-EGFR Nanobodies. Cancer Immunol. Immunother..

[179] Pérez-Martínez D., Infante Y.C., Ramírez B.S., Rojas G. (2022). Domain-level epitope mapping of polyclonal antibodies against HER-1 and HER-2 receptors using phage display technology. Sci. Rep..

[180] Lamtha T., Tabtimmai L., Bangphoomi K., Kiriwan D., Malik A.A., Chaicumpa W., van Bergen En Henegouwen P.M., Choowongkomon K. (2021). Generation of a nanobody against HER2 tyrosine kinase using phage display library screening for HER2-positive breast cancer therapy development. Protein Eng. Des. Sel..

[181] Wang J., Lamolinara A., Conti L., Giangrossi M., Cui L., Morelli M.B., Amantini C., Falconi M., Bartolacci C., Andreani C. (2022). HER2-Displaying M13 Bacteriophages induce Therapeutic Immunity against Breast Cancer. Cancers.

[182] Shadidi M., Sørensen D., Dybwad A., Furset G., Sioud M. (2008). Mucosal vaccination with phage-displayed tumour antigens identified through proteomics-based strategy inhibits the growth and metastasis of 4T1 breast adenocarcinoma. Int. J. Oncol..

[183] Wu Y., Wan Y., Bian J., Zhao J., Jia Z., Zhou L., Zhou W., Tan Y. (2002). Phage display particles expressing tumor-specific antigens induce preventive and therapeutic anti-tumor immunity in murine p815 model. Int. J. Cancer.

[184] Rähni A., Jaago M., Sadam H., Pupina N., Pihlak A., Tuvikene J., Annuk M., Mägi A., Timmusk T., Ghaemmaghami A.M. (2022). Melanoma-specific antigen-associated antitumor antibody reactivity as an immune-related biomarker for targeted immunotherapies. Commun. Med..

[185] Odales J., Servín-Blanco R., Martínez-Cortés F., Valle J.G., Domínguez-Romero A.N., Gevorkian G., Manoutcharian K. (2022). Antitumor efficacy of MUC1-derived variable epitope library treatments in a mouse model of breast cancer. Vaccine.

[186] Mohammadi M., Olsen S.K., Ibrahimi O.A. (2005). Structural basis for fibroblast growth factor receptor activation. Cytokine Growth Factor. Rev..

[187] Dodé C., Levilliers J., Dupont J.-M., De Paepe A., Le Dû N., Soussi-Yanicostas N., Coimbra R.S., Delmaghani S., Compain-Nouaille S., Baverel F. (2003). Loss-of-function mutations in FGFR1 cause autosomal dominant Kallmann syndrome. Nat. Genet..

[188] Kan S.-h., Elanko N., Johnson D., Cornejo-Roldan L., Cook J., Reich E.W., Tomkins S., Verloes A., Twigg S.R., Rannan-Eliya S. (2002). Genomic screening of fibroblast growth-factor receptor 2 reveals a wide spectrum of mutations in patients with syndromic craniosynostosis. Am. J. Hum. Genet..

[189] Webster M.K., Donoghue D.J. (1997). FGFR activation in skeletal disorders: Too much of a good thing. Trends Genet..

[190] Wang J., Stockton D.W., Ittmann M. (2004). The fibroblast growth factor receptor-4 Arg388 allele is associated with prostate cancer initiation and progression. Clin. Cancer Res..

[191] Massari F., Ciccarese C., Santoni M., Lopez-Beltran A., Scarpelli M., Montironi R., Cheng L. (2015). Targeting fibroblast growth factor receptor (FGFR) pathway in renal cell carcinoma. Expert Rev. Anticancer Ther..

[192] Shi S., Li X., You B., Shan Y., Cao X., You Y. (2015). High expression of FGFR4 enhances tumor growth and metastasis in nasopharyngeal carcinoma. J. Cancer.

[193] Rodriguez-Vida A., Saggese M., Hughes S., Rudman S., Chowdhury S., Smith N.R., Lawrence P., Rooney C., Dougherty B., Landers D. (2015). Complexity of FGFR signalling in metastatic urothelial cancer. J. Hematol. Oncol..

[194] Criscitiello C., Esposito A., De Placido S., Curigliano G. (2015). Targeting fibroblast growth factor receptor pathway in breast cancer. Curr. Opin. Oncol..

[195] Wu X., Yan Q., Huang Y., Huang H., Su Z., Xiao J., Zeng Y., Wang Y., Nie C., Yang Y. (2010). Isolation of a novel basic FGF-binding peptide with potent antiangiogenetic activity. J. Cell. Mol. Med..

[196] Wang C., Lin S., Li X., Wu X. (2010). Mechanism of inhibitory effect of P7 on 3T3 cell proliferation induced by basic fibroblast growth factor. Acta Pharmacol. Sin..

[197] Li Q., Gao S., Yu Y., Wang W., Chen X., Wang R., Li T., Wang C., Li X., Wu X. (2012). A novel bFGF antagonist peptide inhibits breast cancer cell growth. Mol. Med. Rep..

[198] Chen Q., Yang Z., Chen X., Shu L., Qu W. (2019). Peptide P7 inhibits the bFGF-stimulated proliferation and invasion of SKOV3 cells. Exp. Ther. Med..

[199] Asadi-Ghalehni M., Rasaee M.J., RajabiBazl M., Khosravani M., Motaghinejad M., Javanmardi M., Khalili S., Modjtahedi H., Sadroddiny E. (2017). A novel recombinant anti-epidermal growth factor receptor peptide vaccine capable of active immunization and reduction of tumor volume in a mouse model. Microbiol. Immunol..

[200] Clark J.R., Bartley K., Jepson C.D., Craik V., March J.B. (2011). Comparison of a bacteriophage-delivered DNA vaccine and a commercially available recombinant protein vaccine against hepatitis B. FEMS Immunol. Med. Microbiol..

[201] Khan K.H. (2013). DNA vaccines: Roles against diseases. Germs.

[202] March J.B., Clark J.R., Jepson C.D. (2004). Genetic immunisation against hepatitis B using whole bacteriophage λ particles. Vaccine.

[203] Coban C., Koyama S., Takeshita F., Akira S., Ishii K.J. (2008). Molecular and cellular mechanisms of DNA vaccines. Hum. Vaccines.

[204] Li L., Saade F., Petrovsky N. (2012). The future of human DNA vaccines. J. Biotechnol..

[205] Hobernik D., Bros M. (2018). DNA vaccines—How far from clinical use?. Int. J. Mol. Sci..

[206] Folegatti P.M., Ewer K.J., Aley P.K., Angus B., Becker S., Belij-Rammerstorfer S., Bellamy D., Bibi S., Bittaye M., Clutterbuck E.A. (2020). Safety and immunogenicity of the ChAdOx1 nCoV-19 vaccine against SARS-CoV-2: A preliminary report of a phase 1/2, single-blind, randomised controlled trial. Lancet.

[207] Nicastro J., Sheldon K., Slavcev R.A. (2014). Bacteriophage lambda display systems: Developments and applications. Appl. Microbiol. Biotechnol..

[208] Clark J.R., March J.B. (2004). Bacteriophage-mediated nucleic acid immunisation. FEMS Immunol. Med. Microbiol..

[209] Ghaemi A., Soleimanjahi H., Gill P., Hassan Z., Jahromi S.R.M., Roohvand F. (2010). Recombinant λ-phage nanobioparticles for tumor therapy in mice models. Genet. Vaccines Ther..

[210] Ghaemi A., Soleimanjahi H., Gill P., Hassan Z.M., Razeghi S., Fazeli M., Razavinikoo S.M.H. (2011). Protection of mice by a λ-based therapeutic vaccine against cancer associated with human papillomavirus type 16. Intervirology.

[211] Thomas B.S., Nishikawa S., Ito K., Chopra P., Sharma N., Evans D.H., Tyrrell D.L.J., Bathe O.F., Rancourt D.E. (2012). Peptide vaccination is superior to genetic vaccination using a recombineered bacteriophage λ subunit vaccine. Vaccine.

[212] Hashemi H., Bamdad T., Jamali A., Pouyanfard S., Mohammadi M.G. (2010). Evaluation of humoral and cellular immune responses against HSV-1 using genetic immunization by filamentous phage particles: A comparative approach to conventional DNA vaccine. J. Virol. Methods.

[213] Ye X., Liu Z., Hemida M.G., Yang D. (2011). Targeted delivery of mutant tolerant anti-coxsackievirus artificial microRNAs using folate conjugated bacteriophage Phi29 pRNA. PLoS ONE.

[214] Bao Q., Li X., Han G., Zhu Y., Mao C., Yang M. (2019). Phage-based vaccines. Adv. Drug. Deliv. Rev..

[215] Kaufmann K.B., Büning H., Galy A., Schambach A., Grez M. (2013). Gene therapy on the move. EMBO Mol. Med..

[216] Ramamoorth M., Narvekar A. (2015). Non viral vectors in gene therapy-an overview. J. Clin. Diagn. Res. JCDR.

[217] Bouard D., Alazard-Dany N., Cosset F.L. (2009). Viral vectors: From virology to transgene expression. Br. J. Pharmacol..

[218] Waehler R., Russell S.J., Curiel D.T. (2007). Engineering targeted viral vectors for gene therapy. Nat. Rev. Genet..

[219] Hosseinidoust Z. (2017). Phage-mediated gene therapy. Curr. Gene Ther..

[220] Hood J.D., Bednarski M., Frausto R., Guccione S., Reisfeld R.A., Xiang R., Cheresh D.A. (2002). Tumor regression by targeted gene delivery to the neovasculature. Science.

[221] Hajitou A., Trepel M., Lilley C.E., Soghomonyan S., Alauddin M.M., Marini F.C., Restel B.H., Ozawa M.G., Moya C.A., Rangel R. (2006). A hybrid vector for ligand-directed tumor targeting and molecular imaging. Cell.

[222] Hajitou A., Lev D.C., Hannay J.A., Korchin B., Staquicini F.I., Soghomonyan S., Alauddin M.M., Benjamin R.S., Pollock R.E., Gelovani J.G. (2008). A preclinical model for predicting drug response in soft-tissue sarcoma with targeted AAVP molecular imaging. Proc. Natl. Acad. Sci. USA.

[223] Przystal J.M., Umukoro E., Stoneham C.A., Yata T., O’Neill K., Syed N., Hajitou A. (2013). Proteasome inhibition in cancer is associated with enhanced tumor targeting by the adeno-associated virus/phage. Mol. Oncol..

[224] Kia A., Przystal J.M., Nianiaris N., Mazarakis N.D., Mintz P.J., Hajitou A. (2012). Dual Systemic Tumor Targeting with Ligand-Directed Phage and Grp78 Promoter Induces Tumor RegressionLigand Systemic Targeting of the Grp78 Promoter. Mol. Cancer Ther..

[225] Smith T.L., Yuan Z., Cardó-Vila M., Sanchez Claros C., Adem A., Cui M.-H., Branch C.A., Gelovani J.G., Libutti S.K., Sidman R.L. (2016). AAVP displaying octreotide for ligand-directed therapeutic transgene delivery in neuroendocrine tumors of the pancreas. Proc. Natl. Acad. Sci. USA.

[226] Chongchai A., Waramit S., Suwan K., Al-Bahrani M., Udomruk S., Phitak T., Kongtawelert P., Pothacharoen P., Hajitou A. (2021). Bacteriophage-mediated therapy of chondrosarcoma by selective delivery of the tumor necrosis factor alpha (TNFα) gene. FASEB J..

[227] Asavarut P., Waramit S., Suwan K., Marais G.J., Chongchai A., Benjathummarak S., Al-Bahrani M., Vila-Gomez P., Williams M., Kongtawelert P. (2022). Systemically targeted cancer immunotherapy and gene delivery using transmorphic particles. EMBO Mol. Med..

[228] Yang Zhou J., Suwan K., Hajitou A. (2020). Initial steps for the development of a phage-mediated gene replacement therapy using CRISPR-Cas9 technology. J. Clin. Med..

[229] Ghosh D., Peng X., Leal J., Mohanty R.P. (2018). Peptides as drug delivery vehicles across biological barriers. J. Pharm. Investig..

[230] Shadidi M., Sioud M. (2003). Identification of novel carrier peptides for the specific delivery of therapeutics into cancer cells. FASEB J..

[231] Fukuta T., Asai T., Kiyokawa Y., Nakada T., Bessyo-Hirashima K., Fukaya N., Hyodo K., Takase K., Kikuchi H., Oku N. (2017). Targeted delivery of anticancer drugs to tumor vessels by use of liposomes modified with a peptide identified by phage biopanning with human endothelial progenitor cells. Int. J. Pharm..

[232] Cai X.-M., Xie H.-L., Liu M.-Z., Zha X.-L. (2008). Inhibition of cell growth and invasion by epidermal growth factor-targeted phagemid particles carrying siRNA against focal adhesion kinase in the presence of hydroxycamptothecin. BMC Biotechnol..

[233] Cohen B.A., Bergkvist M. (2013). Targeted in vitro photodynamic therapy via aptamer-labeled, porphyrin-loaded virus capsids. J. Photochem. Photobiol. B Biol..

[234] Gandra N., Abbineni G., Qu X., Huai Y., Wang L., Mao C. (2013). Bacteriophage bionanowire as a carrier for both cancer-targeting peptides and photosensitizers and its use in selective cancer cell killing by photodynamic therapy. Small.

[235] Dong X., Pan P., Zheng D.-W., Bao P., Zeng X., Zhang X.-Z. (2020). Bioinorganic hybrid bacteriophage for modulation of intestinal microbiota to remodel tumor-immune microenvironment against colorectal cancer. Sci. Adv..

[236] Maji M., Mazumder S., Bhattacharya S., Choudhury S.T., Sabur A., Shadab M., Bhattacharya P., Ali N. (2016). A lipid based antigen delivery system efficiently facilitates MHC class-I antigen presentation in dendritic cells to stimulate CD8+ T cells. Sci. Rep..

[237] Wan Y., Wu Y., Bian J., Wang X., Zhou W., Jia Z., Tan Y., Zhou L. (2001). Induction of hepatitis B virus-specific cytotoxic T lymphocytes response in vivo by filamentous phage display vaccine. Vaccine.

[238] Eriksson F., Tsagozis P., Lundberg K., Parsa R., Mangsbo S.M., Persson M.A., Harris R.A., Pisa P. (2009). Tumor-specific bacteriophages induce tumor destruction through activation of tumor-associated macrophages. J. Immunol..

[239] Matsuzaki S., Uchiyama J., Takemura-Uchiyama I., Daibata M. (2014). Perspective: The age of the phage. Nature.

[240] Young R., Gill J.J. (2015). Phage therapy redux—What is to be done?. Science.

[241] Ledford H. (2015). Cancer-fighting viruses near market. Nature.

[242] Roehnisch T., Then C., Nagel W., Blumenthal C., Braciak T., Donzeau M., Böhm T., Flaig M., Bourquin C., Oduncu F.S. (2014). Phage idiotype vaccination: First phase I/II clinical trial in patients with multiple myeloma. J. Transl. Med..

[243] Dąbrowska K. (2019). Phage therapy: What factors shape phage pharmacokinetics and bioavailability? Systematic and critical review. Med. Res. Rev..

[244] Veeranarayanan S., Azam A.H., Kiga K., Watanabe S., Cui L. (2022). Bacteriophages as solid tumor theragnostic agents. Int. J. Mol. Sci..

[245] Yao V.J., Ozawa M.G., Trepel M., Arap W., McDonald D.M., Pasqualini R. (2005). Targeting pancreatic islets with phage display assisted by laser pressure catapult microdissection. Am. J. Pathol..

[246] Morrison D., Ulevitch R. (1978). The effects of bacterial endotoxins on host mediation systems. A review. Am. J. Pathol..

[247] Liu D., Van Belleghem J.D., de Vries C.R., Burgener E., Chen Q., Manasherob R., Aronson J.R., Amanatullah D.F., Tamma P.D., Suh G.A. (2021). The safety and toxicity of phage therapy: A review of animal and clinical studies. Viruses.

[248] Bordier C. (1981). Phase separation of integral membrane proteins in Triton X-114 solution. J. Biol. Chem..

[249] Szermer-Olearnik B., Boratyński J. (2015). Removal of endotoxins from bacteriophage preparations by extraction with organic solvents. PLoS ONE.

